# Integrative Metabolomic and Agronomic Analyses Reveal the Impact of *Pseudomonas fluorescens*-Based Bioinputs on the Micro-Tom Tomato (*Solanum lycopersicum*) Model System

**DOI:** 10.1021/acsomega.6c04589

**Published:** 2026-09-14

**Authors:** Stephanie Nemesio da Silva, Rodrigo Alberto Repke, Lázaro Eustaquio Pereira Peres, Alice Aranda Peres, Rafael Costa Santos Rocha, Danilo da Cruz Centeno, Johana Rincones Pérez, Taicia Pacheco Fill

**Affiliations:** † Institute of Chemistry, 344102 University of Campinas, Campinas, São Paulo 13083-970, Brazil; ‡ Rovensa Next, Hortolândia, São Paulo 13186-904, Brazil; § Department of Biological Sciences, Escola Superior de Agricultura “Luiz de Queiroz”, 54538 University of São Paulo, Piracicaba, São Paulo 13418-900, Brazil; ∥ Center of Natural and Human Sciences, Federal University of ABC, São Bernardo do Campo, São Paulo 09606-045, Brazil

## Abstract

Microbial biostimulants, bioinputs based on microorganisms, have emerged as an alternative to increase crop yield and quality while reducing the environmental impact. Particularly, the plant growth-promoting bacterium *Pseudomonas fluorescens* exhibits high potential for application in this field due to its production of a range of specialized metabolites (SMs). Research in this area mainly focuses on the use of active bacterial cells, whose efficacy depends on the success of host colonization. In this regard, we evaluated the treatment effect of two bioinputs from *P. fluorescens* ATCC_13525 (with active and inactive bacterial cells) on Micro-Tom tomato growth, productivity, nutrient accumulation, and metabolism. The bioinput with active bacterial cells promoted a few beneficial effects on the plant, the main one being associated with an increase in the calcium (Ca) concentration in fruits through foliar application. The cell-free bioinput primarily promoted an increase in leaf area, root biomass, and phosphorus (P) and molybdenum (Mo) concentrations in fruits. Specifically, its application by the foliar method exhibited a balance between the Brix degree and the maintenance of productivity. Untargeted metabolomic analysis of tomato leaves treated with this method indicated modulation of phenolamides and glycerophospholipids. On the other hand, an increase in the abundance of tryptophan and the indole lycoperodine-1 occurred when the treatment was carried out via soil. The results highlight that the cell-free formulation modulates plant responses and enhances plant performance in certain aspects under the evaluated application conditions, suggesting an important biostimulant effect of the metabolites present in the cell-free microbial culture for growth promotion and improvement in fruit quality. Notably, the findings demonstrate that microbial metabolites alone can drive significant plant responses, opening new perspectives for next-generation bioinputs in sustainable agriculture.

## Graphical abstract

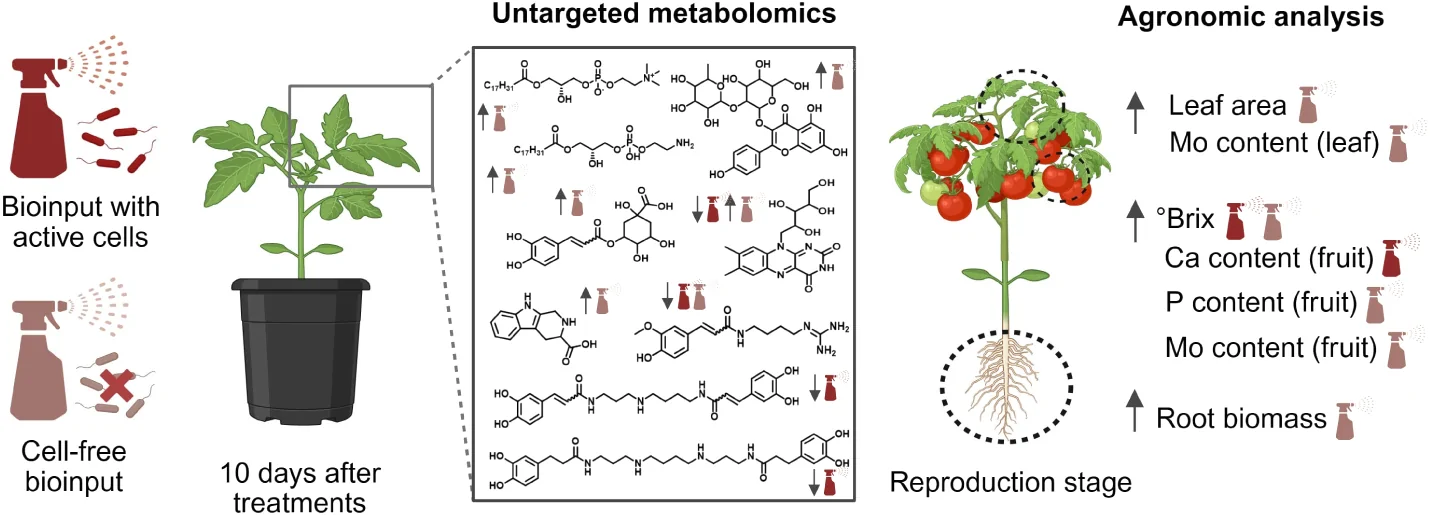

## Introduction

Given the urgent need to protect and enhance the natural resource base while improving crop productivity and quality, more sustainable agricultural practices and technologies have emerged. These include biostimulants that have gained popularity in recent years.^1^ This is a growing sector valued at US$4.46 billion in 2025 and projected to reach US$7.29 billion by 2030.^2^ Biostimulants can be composed of a wide range of substances originating from different biological sources, whose primary function is to stimulate natural physiological processes that promote plant growth and development.^3,4^ Among the existing types, microbial biostimulants have gained significant attention worldwide. Obtained from beneficial bacteria and fungi, they exhibit high metabolic complexity that promotes crop physiological responses through synergistic effects.^1,5^

*Pseudomonas fluorescens*, a Gram-negative environmental bacterium, has stood out because of its high biotechnological potential in agricultural applications. The species is characterized by its genetic and metabolic diversity and is capable of producing a wide range of bioactive compounds that can modulate nutrient availability, influence plant physiological responses, and contribute to plant growth promotion.^6,7^ Research and agricultural applications involving *P. fluorescens* have focused on formulations containing viable bacterial cells, whose beneficial effects on the host plant are associated with successful rhizosphere colonization.^8^ However, colonization efficiency is strongly related to bacterial viability, which can be negatively affected by factors such as storage time, humidity, and temperature variations.^9^ Therefore, these factors can potentially reduce or compromise the formulation effectiveness.

In this context, cell-free microbial culture formulations have emerged as promising alternatives due to their improved stability during storage and handling.^10^ Beyond extending shelf life, facilitating transport, and enabling large-scale agricultural application, these formulations may also offer advantages related to product standardization, reduced biosafety concerns, and potentially less complex regulatory requirements.^11^ Furthermore, comparing formulations containing active and inactive bacterial cells may help distinguish plant responses associated with bacterial colonization from those mediated by microbial metabolites. Nevertheless, studies directly comparing bioinputs derived from the same bacterial strain but differing specifically in cellular viability are still scarce, limiting our understanding of the mechanisms underlying plant responses to microbial-based products.

Model plants are commonly used to achieve a systematic understanding of agricultural products. An important model for research related to specialized metabolism and fruit development is the tomato (*Solanum lycopersicum*).^12^ Among tomato varieties, the cultivar Micro-Tom (MT) has been extensively used because it has advantages, including its small size, short life cycle, and capacity to grow under artificial light at high density.^13^ Therefore, MT can provide new insights into metabolic processes and productivity associated with microbial inputs.

In this study, we aimed to investigate how two bioinputs obtained from *Pseudomonas fluorescens* ATCC_13525, differing in cell viability (active and inactive bacterial cells), affect plant physiological aspects under controlled cultivation conditions using the Micro-Tom model system. To address this, we combined the microbial metabolic characterization of the formulations with plant phenotypic, metabolomic, productivity, and fruit quality analyses to better understand the differential responses associated with active bacterial colonization and/or metabolites present in the bioinputs.

## Results and Discussion

### Specialized Metabolites Produced by the Strain Detected in the Bioinputs

*P. fluorescens* is well-recognized for its ability to produce a wide range of bioactive compounds that contribute directly to plant health and growth promotion. Investigating the metabolic differences among bioinputs derived from this strain can therefore provide valuable insights into the specific mechanisms underlying each product’s activity. To address this, we employed an untargeted metabolomics approach based on LC–HRMS. Initially, the unsupervised exploratory Principal Component Analysis (PCA) method was used to assess trends among sample groups and quality control (QC) patterns. The PCA scores plot of the first two principal components (Figure 1A) exhibited a clear separation between bioinputs with active (P_active) and inactive (P_inactive) bacterial cells, with samples located in the positive quadrant of PC1 and varying along PC2. The tight clustering of QCs indicates stable performance of the instrument.

**Figure 1:**
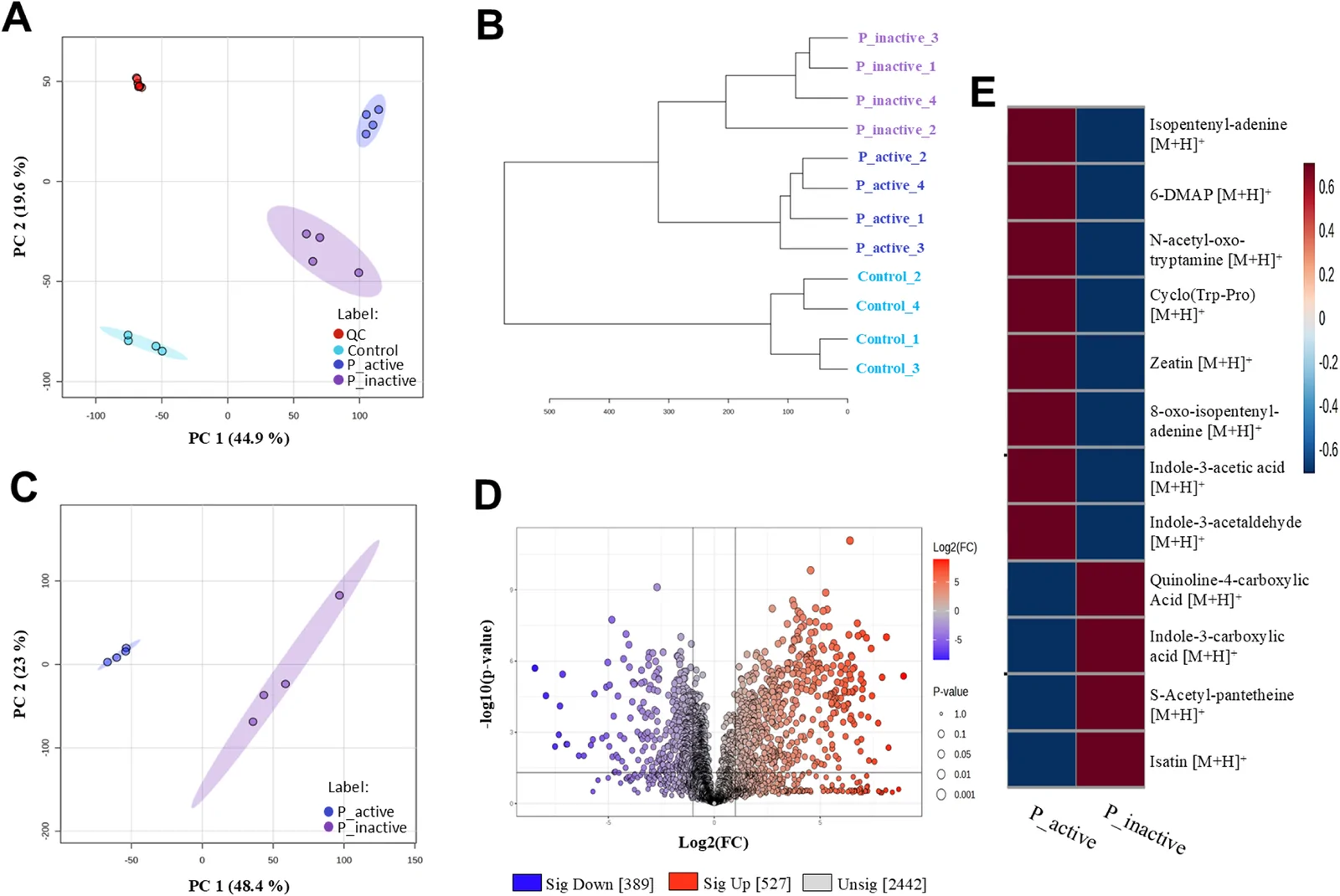
(A) PCA scores plot with all groups, (B) HCA dendrogram, (C) PCA scores plot with only bioinputs samples, (D) volcano plot with the direction of comparison: P_active/P_inactive, and (E) heatmap of differential metabolites obtained from LC–HRMS data of *P. fluorescens* bioinputs.

Through hierarchical cluster analysis (HCA), a tree diagram was used to display the relationship between samples. As shown in Figure 1B, the samples were grouped into two clusters: culture medium (control) and bioinputs. In turn, the bioinput cluster was segmented into P_active and P_inactive, corroborating the PCA results. The data indicate significant differences in the metabolic profiles of the products. The PCA method applied to P_active and P_inactive groups (Figure 1C) indicated greater variability between the P_inactive samples. The combination of fold change (FC) and *t*-test analyses through a volcano plot (Figure 1D) indicated that the majority of features with statistical/biological significance increased their content in P_inactive, denoted as Sig Up.

Based on *p*-value ≤ 0.05, VIP ≥ 0.6, and FC > 2 or < 0.5 as selection criteria, 12 differential microbial metabolites among the bioinputs were annotated based on MS/MS fragmentation pattern comparisons with the literature and/or public spectral databases, using in silico analysis by SIRIUS as an auxiliary tool to support initial candidate formula and structure prediction. The metabolites are listed in Table S1 by chemical class. The significant differences in the abundance of these metabolites between the bioinputs were visualized using a heatmap obtained through HCA, showing only group averages. As shown in Figure 1E, 8 metabolites were more abundant in the product with active bacterial cells, while the other 4 were more abundant in the product with inactive bacterial cells.

Among the most abundant metabolites in P_active are three aminopurines: isopentenyladenine (iP, [M + H]^+^ at *m*/*z* 204.1244), zeatin [M + H]^+^ at *m*/*z* 220.1189, and 8-oxo-isopentenyladenine (8-oxo-iP, [M + H]^+^ at *m*/*z* 220.1190); one purine: 6-dimethylaminopurine (6-DMAP, [M + H]^+^ at *m*/*z* 164.0931); three indole compounds: indole-3-acetaldehyde (IAL, [M + H]^+^ at *m*/*z* 160.0754), indole-3-acetic acid (IAA, [M + H]^+^ at *m*/*z* 176.0704), and N-acetyl-oxotryptamine [M + H]^+^ at *m*/*z* 217.0968; and the diketopiperazine cyclo­(Trp-Pro) [M + H]^+^ at *m*/*z* 284.1389. On the other hand, P_inactive exhibited a greater abundance of quinoline-4-carboxylic acid (Q4CA, [M + H]^+^ at *m*/*z* 174.0548); two indoles: isatin [M + H]^+^ at *m*/*z* 148.0390 and indole-3-carboxylic acid [M + H]^+^ at *m*/*z* 162.0547; and the alkylthiol acetyl-pantetheine [M + H]^+^ at *m*/*z* 321.1474.

*P. fluorescens* is known to produce cytokinins, including isopentenyladenine and zeatin-type compounds,^14^ indole auxins,^15^ quinolines,^16^ and diketopiperazines,^17^ but the metabolites 8-oxo-isopentenyladenine and cyclo­(Trp-Pro) have not been described for the species to date. After data analysis using the microbeMASST taxonomic tool on the GNPS platform,^18^ it was possible to detect these compounds in *P. fluorescens* samples deposited in the GNPS database. The microbeMASST results, the experimental MS/MS spectra used in the search, and the data set references are shown in Figure S1. Thus, although these metabolites have not yet been previously reported for the species, LC–MS public spectral repositories provide supporting evidence for their occurrence in *P. fluorescens*. The detection of these compounds expands the current knowledge regarding the bacteria’s metabolic repertoire and highlights the biosynthetic capacity of the species.

Often, the growth-promoting effect of *P. fluorescens* has been associated with plant hormone production,^19^ demonstrating that IAA secretion by the strain during interaction with *S. lycopersicum* can improve seed germination, root and shoot length, and biomass of tomato seedlings. In fact, 9 of the 12 specialized differential metabolites found are plant hormones or plant hormone intermediates: isatin, IAA, IAL, indole-3-carboxylic acid, N-acetyl-oxotryptamine, zeatin, iP, 8-oxo-iP, and 6-DMAP. They are directly or indirectly involved in essential processes in the plant, including stimulation of cell division, regulation of growth, and flowering.^20^ The bacterium is also known to produce antimicrobial compounds that can act directly in pathogen suppression or induce plant defense and stress responses, interfering with the quorum-sensing process and contributing to the health and maintenance of the plant. Two metabolites with antimicrobial activity described in the literature appear as differentiating factors between the bioinputs: cyclo­(Trp-Pro), with a greater abundance of P_active, and Q4CA, more abundant in P_inactive.^21,22^ S-Acetyl-pantetheine, a derivative of pantothenate/vitamin B5, is the only differential metabolite that does not have a biological function known in plants yet. As an acetyl-CoA precursor, it provides the essential acetyl group for vital plant metabolic processes, including energy production and lipid synthesis,^23^ but further studies are needed to elucidate its biological role upon exogenous application.

### Treatment Effects on Plant Growth, Water-Use Efficiency, Photosynthesis, and Productivity

The effects of the bioinputs on the development, maintenance, and productivity of plants were evaluated by using the Micro-Tom tomato plant model. Both soil and foliar applications were performed according to the experimental scheme shown in Figure 2A. Plant height, measured between 4 and 25 days post-treatment, exhibited statistically significant variations compared to the control T1 (Figure 2B). Foliar application of the bioinput containing active bacterial cells (T3) resulted in a reduction in plant height between 8 and 16 days, whereas both treatments with the product containing inactive bacterial cells (T4 and T5) promoted a slight increase in plant height 8 days after treatment. P_inactive also stimulated root growth, exhibiting an increase in fresh root mass of up to 57% (Figure 2C) and dry root mass of 26% (Figure 2D).

**Figure 2:**
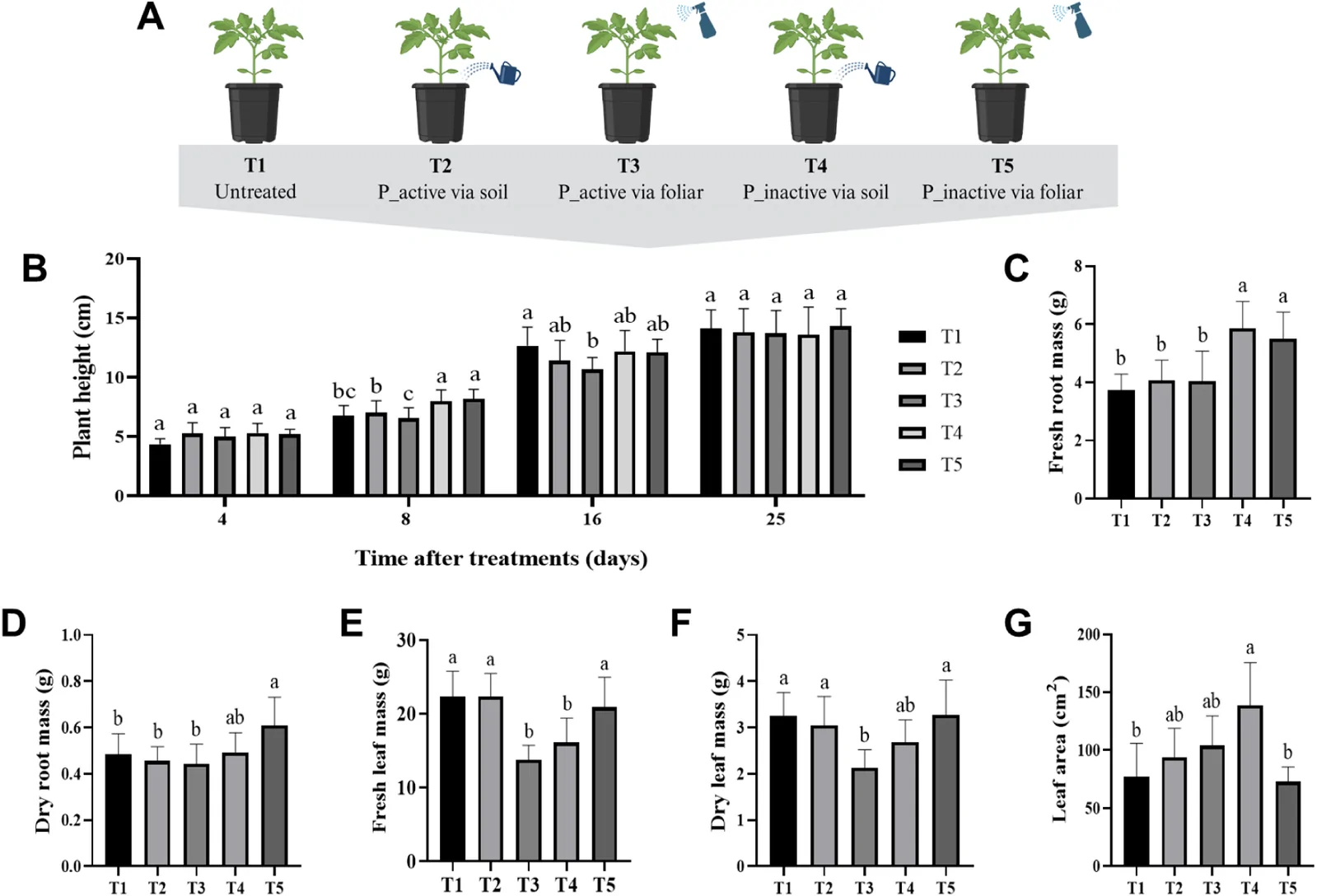
(A) Experimental scheme used for the treatment of Micro-Tom tomato with bioinputs from *P. fluorescens*. Phenological data of (B) plant height, (C) fresh root mass, (D) dry root mass, (E) fresh leaf mass, (F) dry leaf mass, and (G) leaf area. Error bars represent the SD. Different letters indicate significantly different values at *p* ≤ 0.05 (one-way ANOVA, Tukey’s posthoc test).

On the other hand, there was no observed increase in fresh and dry leaf masses relative to the control group. Contrarily, plants in T3 and those treated with the bioinput with inactive bacterial cells via soil (T4) presented a reduction in leaf fresh weight (Figure 2E). However, only T3 showed a significant decrease in leaf dry weight (Figure 2F). Curiously, T4 led to a significant increase in leaf area by 80% (Figure 2G), exhibiting an inverse relationship between the size and biomass of the leaves in this treatment. Although apparently counterintuitive at first glance, this may indicate the formation of thinner leaves with lower tissue density. Complementary analyses of leaf micro and macronutrient concentrations (Figure S2) revealed significant variation only for molybdenum (Mo), with T4 showing a 68% increase compared with the control treatment, reaching a concentration of 1.31 mg/kg.

Reported benefits of *P. fluorescens*-based bioinputs include an increase in plant height and root development,^24,25^ often related to the production of plant hormones by the bacteria.^26–28^ According to our results, the growth effects were observed mainly in P_inactive. Despite the greater abundance of plant hormones in P_active (Figure 1E), the cell-free product (P_inactive) elicited a more effective plant growth response, likely because it provides direct contact between metabolites and plant tissue, avoiding the variability inherent to the colonization of live microbial bioinputs.^29^

Improvements in root measures by *P. fluorescens* are commonly described after soil or seed treatment with the microbial inoculant,^30,31^ considering that bacterial colonization primarily occurs in the plant rhizosphere.^32^ However, no significant difference in root biomass was observed with P_active when applied to either the soil or the leaves. Only treatments with P_inactive improved root growth, indicating that the stimulation of the root system may be associated with the metabolite components rather than the active cells. Particularly, foliar application of P_inactive increased the fresh and dry weight of Micro-Tom roots. Similar findings were previously reported for *Megathyrsus maximus* after leaf spray of *P. fluorescens* CNPSo 2799, leading to a 31% increase in dry root weight.^33^ This effect can be attributed to systemic signaling communication from shoots to roots, which involves alterations in the plant’s hormone levels triggered by external stimuli.^34,35^

*P. fluorescens* is well known to improve plant nutrient uptake. Seed and leaf spray inoculation with *P. fluorescens* CNPSo 2719 in brachiarias (*Urochloa* spp.) increased P content by 33.3% and 36.6% and K by 10.6% and 13.6%, respectively.^36^ In sweet cherry (*Prunus avium*), floral and foliar applications of *P. fluorescens* BA-8 significantly enhanced the uptake of K, Ca, Mg, Fe, Mn, and Zn in leaves.^37^ Although many studies report changes in nutrient acquisition using active cells, there is a gap in understanding the nutritional effects of cell-free extracts. According to our analysis, plants treated with P_inactive via soil showed a significant increase in the Mo concentration in Micro-Tom leaves compared with the control (Figure S2). Initially, this result could be attributed to the decrease in leaf biomass in T4 (as shown in Figure 2E), which would promote a nutrient concentration effect even without an increase in Mo uptake. In this case, T3 would also exhibit a similar effect. However, there was no increase in the Mo concentration in T3 despite the decrease in leaf biomass. This suggests that the response observed in T4 may not be explained by a concentration effect but rather a change in Mo uptake. To allow direct inference, further studies evaluating direct measurements of total Mo content per plant and/or isotopic labeling approaches are necessary.

Mo is an essential nutrient that acts as a cofactor for key enzymes involved in several physiological processes, especially nitrogen metabolism.^38^ Higher Mo concentration stimulates enzyme activity, leading to more efficient N assimilation and fixation, thereby improving crop growth.^39^ Among the physiological benefits reported for Mo application, improvement in leaf area has been described.^40^ This suggests that an increase in Mo concentration can be related to the increase in leaf area observed in T4. Although the Mo content is rarely evaluated in studies of bioinputs, effects on leaf area have been described following application of *P. fluorescens*. Increased leaf area has been reported in two varieties of wheat (*Triticum aestivum*) after inoculation with strain FP2 and salicylic acid,^41^ blueberry (*Vaccinium myrtillus*) after soil treatment using *P. fluorescens* Pf5,^42^ and the ornamental plant *Pelargonium peltatum* through *P. fluorescens* strain 148 applied to the soil.^43^

Significant differences in gas-exchange parameters were detected only 23 days after treatment. All applications of the bioinputs exhibited a decrease in the net photosynthetic rate (A, Figure 3A), while stomatal conductance showed a decrease only in T2 (gs, Figure 3B). There were no significant changes in the transpiration rate (E, Figure 3C) and intercellular CO_2_ concentration (Ci, Figure 3D) at the evaluated times. Similarly, no changes in intrinsic water-use efficiency (iWUE) were detected (Figure S3). The reduction in A was not accompanied by significant changes in E, Ci, or iWUE, indicating that the observed response was unlikely to result from stomatal limitation. Instead, it may reflect transient biochemical limitations, such as changes in the efficiency of carbon fixation or other processes associated with the photosynthetic machinery.^44^ Furthermore, the maintenance of transpiration and iWUE suggests that the bioinputs did not compromise the plant’s water balance.

**Figure 3:**
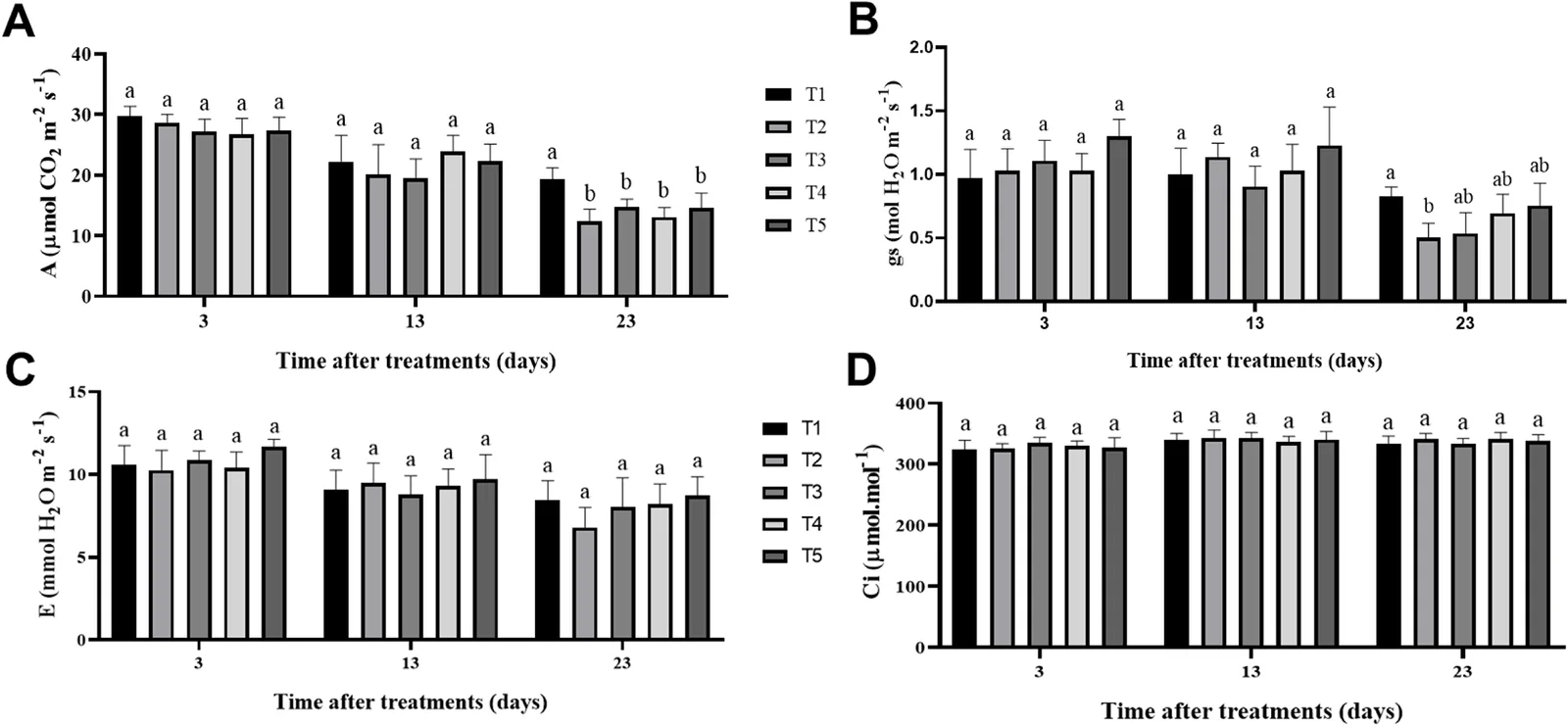
Gas exchange parameters of Micro-Tom tomato treated with *P. fluorescens*-based bioinputs: (A) net photosynthetic rate “A”, (B) stomatal conductance “gs”, (C) transpiration rate “E”, and (D) intercellular CO_2_ concentration “Ci”. Error bars represent the SD. Different letters indicate significantly different values at *p* ≤ 0.05 (one-way ANOVA, Tukey’s posthoc test).

Productivity-related parameters were also evaluated. Figure 4A presents a representation of the harvest obtained for each treatment. Compared to the control, the number of ripe fruits per plant decreased significantly in all treatments applied (Figure 4B), as well as the mass of green and ripe fruits per plant (Figure 4C). When measuring only the mass of ripe fruits, all treatments exhibited a significant decrease, except for T5 (Figure 4D). For the average mass of ripe fruits (Figure 4E), there were no significant changes. These results indicate that both bioinputs affected reproductive parameters, reducing the number of ripe fruits without substantially affecting the fruit size. Although direct phytotoxic effects caused by the production of phytotoxic metabolites could be considered a possible contributing factor,^45,46^ no visible symptoms of damage were observed (Figure S4). Therefore, the reduction in productivity does not appear to be associated with evident phytotoxic effects under evaluated conditions.

**Figure 4:**
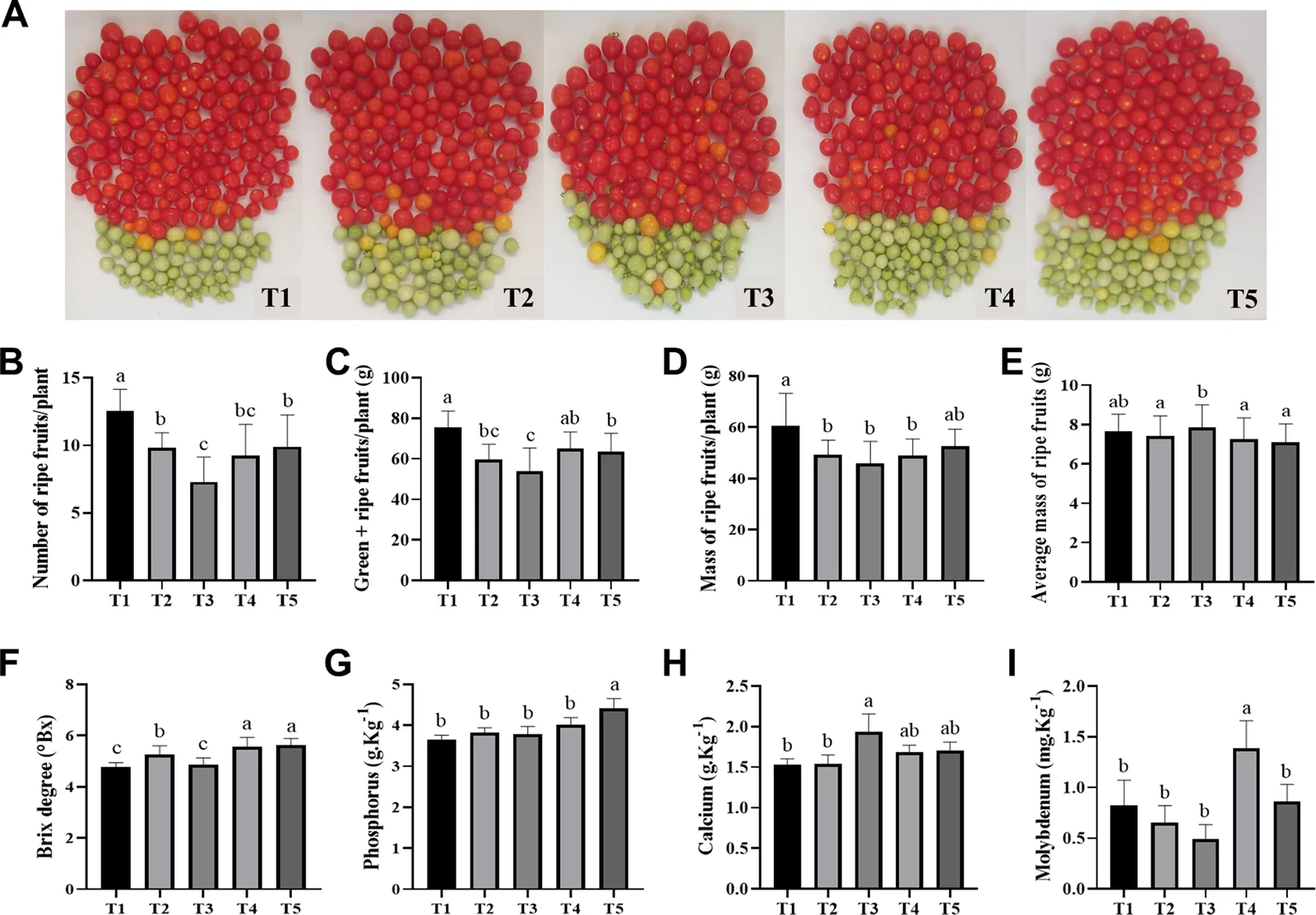
(A) Representation of fruit harvest by treatment, (B) number of ripe fruits/plant, (C) mass of green and ripe fruits/plant, (D) mass of ripe fruits/plant, (E) average mass of ripe fruits, (F) Brix degree, nutrients in the fruit: (G) phosphorus, (H) calcium, and (I) molybdenum. Error bars represent the SD. Different letters indicate significantly different values at *p* ≤ 0.05 (one-way ANOVA, Tukey’s posthoc test).

Plant responses to microbial bioinputs may involve complex physiological and metabolic adjustments, including shifts in hormonal balance, changes in resource allocation, and specialized metabolism, potentially resulting in distinct trade-offs among growth, defense responses, nutrient allocation, and reproductive performance.^47,48^ Therefore, no mechanistic relationship can be established based on the current data, and further targeted analyses would be necessary to clarify the processes underlying the reduction in fruit productivity. Plant metabolic alterations associated with the treatments are further discussed in the topic addressing plant metabolic responses to treatments with bioinputs.

A fundamental quality parameter in tomato fruits is the soluble solids content, measured in Brix degree (°Brix). As shown in Figure 4F, all treatments showed higher °Brix values than the control, except for T3. Similar effects have previously been reported in tomatoes after inoculation with different strains of *P. fluorescens*.^49,50^ Interestingly, treatments T2, T4, and T5 exhibited an inverse relationship between °Brix and productivity, in which reductions in fruit number and total fruit mass per plant were accompanied by increased soluble solids content. Therefore, the decrease in the number of fruits produced may favor the quality of the remaining ones. Because °Brix is commonly associated with sweetness and flavor-related attributes in fruits, the observed increase in °Brix may indicate a potential improvement in sensory quality. For that, specific studies are required. Among the evaluated treatments, T5 was particularly noteworthy because the reduction in fruit mass was not statistically significant, while the increase in °Brix was the highest observed, reaching up to 18%. This suggests a balance between increasing °Brix and maintaining productivity with this application method under the evaluated conditions, resulting in a reasonable production of higher-quality fruit.

Additionally, fruit macronutrient analysis revealed a significant 21% increase in phosphorus concentration in T5, reaching 4.42 g/kg, which corresponded to a 21% increase relative to the control treatment (Figure 4G), while calcium concentration increased exclusively in T3, reaching 1.93 g/kg, corresponding to a 26% increase compared with the control (Figure 4H). Regarding micronutrient analysis, the molybdenum concentration increased by 68% in T4 compared with the control, reaching 1.39 g/kg (Figure 4I), suggesting efficient nutrient transport from vegetative tissues to reproductive organs. The other macro- and micronutrients analyzed are shown in Figure S5.

Phosphorus and calcium accumulations are associated with several plant benefits, including fruit quality.^51^ Previous studies have demonstrated the maintenance of firmness and improvement of the shelf life of tomato fruits after Ca application,^52^ in addition to increasing soluble sugars through P supply.^53^ Calcium is an important aspect in fruit health, considering that its deficiency is associated with blossom-end rot (BER), a devastating physiological disorder that compromises the yield and quality of the vegetable worldwide.^54,55^ In severe cases, BER can result in production losses that reach up to 80% of the total yield.^56^ Although studies have shown that *P. fluorescens* plays a significant role in nutrient uptake, an increase in fruit calcium content has not yet been reported for applications of the bacteria to the best of our knowledge.

Based on the agronomic results, it can be inferred that treatments with the cell-free bioinput (T4 and T5) were more closely associated with phenological improvements and fruit quality parameters under the evaluated application conditions. They include an increase in plant height, root development, and °Brix values. As expected, the type of application method promoted considerable differences related to the control, depending on the parameter evaluated. Soil application promoted an increase in leaf area and Mo concentrations in fruits and leaves, while foliar application increased root biomass and phosphorus concentration in fruits and exhibited a putative trade-off between °Brix and productivity. Additionally, the product with active *P. fluorescens* cells applied via foliar (T3) was the only treatment that increased the concentration of calcium in fruits, an important nutrient for tomato fruit health. Thus, these three treatments exhibit potential to improve crop health, growth, and quality through different action modes.

### Plant Metabolic Responses to Treatments with the Bioinputs

To investigate the established systemic metabolic state in Micro-Tom plants resulting from treatments T3–T5, untargeted metabolomics analyses were applied through LC–HRMS on the first fully expanded leaf after 10 days of treatment. The score plot of the unsupervised PCA (Figure 5A) did not exhibit a separation between the treatments, highlighting the high similarity between the metabolic profiles. Through the supervised method Partial Least Squares Discriminant Analysis (PLS-DA), significant features were visualized by the Variable Importance in Projection (VIP), with values ≥0.6 as a selection criterion. The robustness of the PLS-DA model was supported by 5-fold cross-validation (*R* ^2^ = 0.80, *Q* ^2^ = 0.57) and permutation test (*n* = 1000), indicating the high reliability of the model. According to the VIP scores table in Figure 5B, among the 35 most important features in the projection were tryptophan [M + H]^+^ at *m*/*z* 205.0971, chlorogenic acid [M + H]^+^ at *m*/*z* 355.1021, kaempferol 3-neohesperidoside [M + H]^+^ at *m*/*z* 595.1648, N-caffeoylputrescine [M + H]^+^ at *m*/*z* 251.1389, feruloylagmatine [M + H]^+^ at *m*/*z* 307.1763, and LPE 18:2 [M + H]^+^ at *m*/*z* 478.2927. Particularly, abundance levels of N-caffeoylputrescine were significantly modified only in T3 and T4 (Figure 5C). On the other hand, the amino acid tryptophan and its tetrahydro-β-carboline alkaloid derivative lycoperodine-1 [M + H]^+^ at *m*/*z* 217.0971 showed accumulation in T4 (Figure 5D). The annotated metabolites are listed in Table S2.

**Figure 5:**
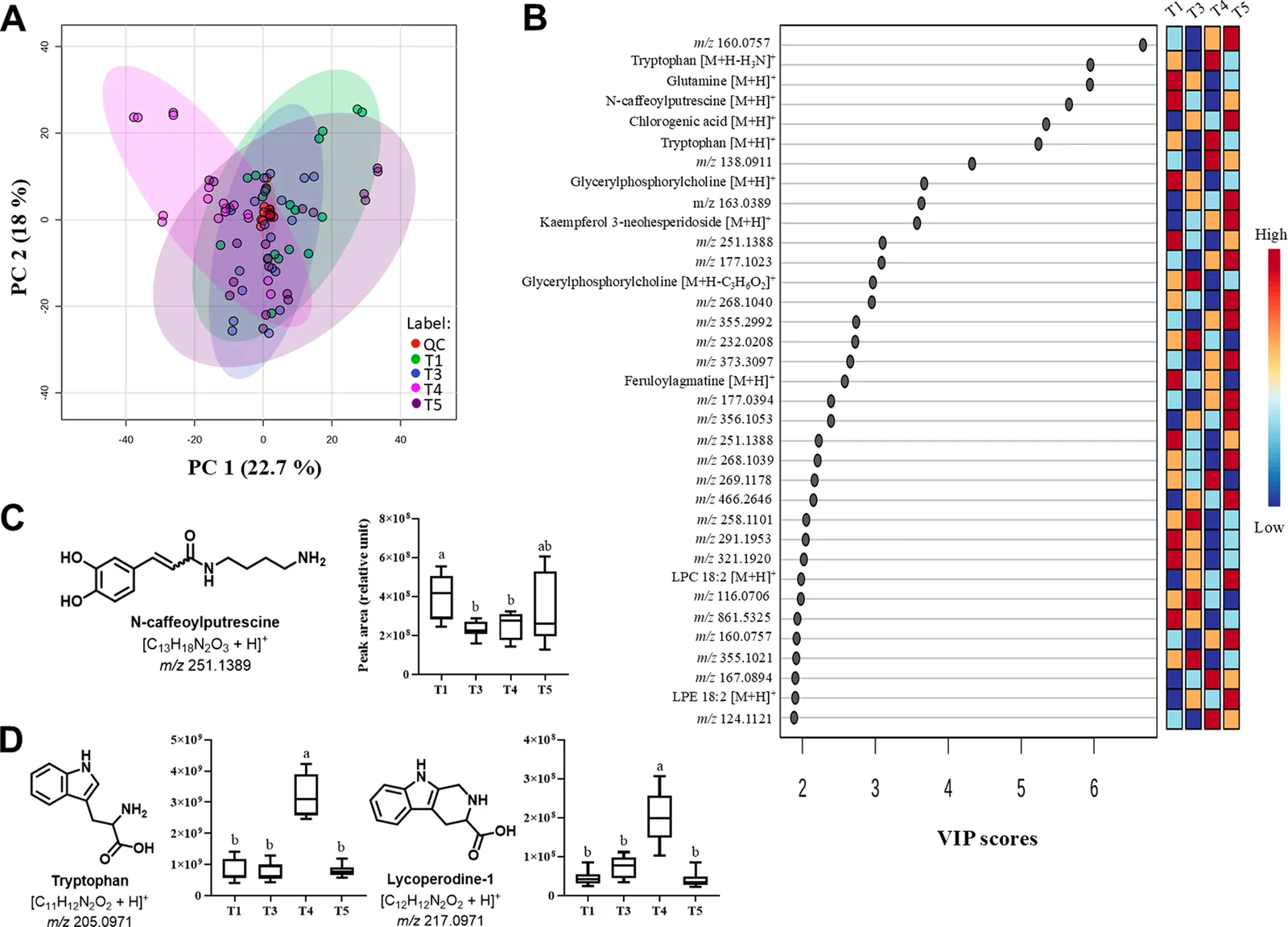
(A) PCA scores plot, (B) VIP scores table, relative abundance of (C) N-caffeoylputrescine, (D) tryptophan and indole derivative boxplot from LC–HRMS data for tomato plants treated with *P. fluorescens* bioinputs. Different letters indicate significantly different values at *p* ≤ 0.05 (one-way ANOVA, Tukey’s posthoc test).

The increase in tryptophan and lycoperodine-1 abundance only in T4 indicates that applying P_inactive via soil modulates the tryptophan pathway and activates the biosynthesis of specific indoles in the plant. Indoles are involved in several plant processes and play a central role in plant growth, signaling, and nutrient absorption.^57^ According to the agronomic results shown in Figure 2, T4 exhibited an initial positive effect on plant height, with a significant difference compared to the control 8 days after treatment. However, this effect was not maintained in the subsequent evaluations. Furthermore, T4 and T5 promoted a significant increase in fresh root mass, while T4 was the only treatment associated with an increase in leaf area. Thus, the effects observed in T4 may be related to the modulation of indole metabolism, as evidenced by the accumulation of tryptophan and lycoperodine-1 in this treatment.

Furthermore, treatment with P_inactive via soil was the only treatment to exhibit Mo accumulation in both leaves and fruits. As a micronutrient involved in key enzymatic processes related to nitrogen metabolism and phytohormone biosynthesis, changes in the Mo concentration may influence plant metabolic regulation.^58^ Previous studies have shown that molybdenum excess can alter the metabolic profile of soybean (*Glycine max* L.) seedlings, including the accumulation of hormones and hormone derivatives.^59^ Therefore, the increase in Mo concentration observed in T4 may be associated with the modulation of indole compounds in this treatment. However, further targeted assays are required to support a causal interpretation of this relationship.

Kaempferol 3-neohesperidoside [M + H]^+^ is a flavonol glycoside whose biological function is commonly related to the stress response, acting as an antioxidant agent.^60^ The increase in the levels of this metabolite is observed only in treatments with the cell-free bioinput, indicating that the application of the product with active bacterial cells does not trigger certain well-known response mechanisms that mitigate oxidative stress involving the biosynthesis of flavonoids. In this case, the stimulating effect of P_active on the plant tends to be smaller than that of P_inactive. In fact, the results of the agronomic measurements corroborate this hypothesis.

LPE 18:2 [M + H]^+^ refers to lysophosphatidylethanolamine with an 18-carbon fatty acid chain containing two double bonds, specified as linoleoyl. Like other molecular species of LPE (e.g., 16:1, 18:1, and 18:3), LPE 18:2 is a component of the plant cell membrane, whose levels can change under stress conditions,^61^ and this molecule can induce the activity of enzymes linked to the defense response.^62^ Thus, T5 can trigger biochemical mechanisms in the plant that are associated with an increase in LPE 18:2 content.

Using the Feature-Based Molecular Networking (FBMN) computational tool, a spectral family of phenolamides and hydroxycinnamate-related metabolites was obtained, including the discriminant metabolites N-caffeoylputrescine, feruloylagmatine, and chlorogenic acid with VIP ≥ 0.6 (Figure 6A). Particularly, chlorogenic acid [M + H]^+^, [M + NH_4_]^+^, [M + K]^+^, and coumaroylquinic acid [M + H]^+^ exhibited accumulation in T3 and T5, while paprazine [M + H]^+^ showed decreased levels in all treatments with P_inactive (T4 and T5). These results provide evidence of significant changes in the phenolamide biosynthetic pathway in all treatments evaluated, involving accumulation or decrease in their levels depending on the treatment performed.

**Figure 6:**
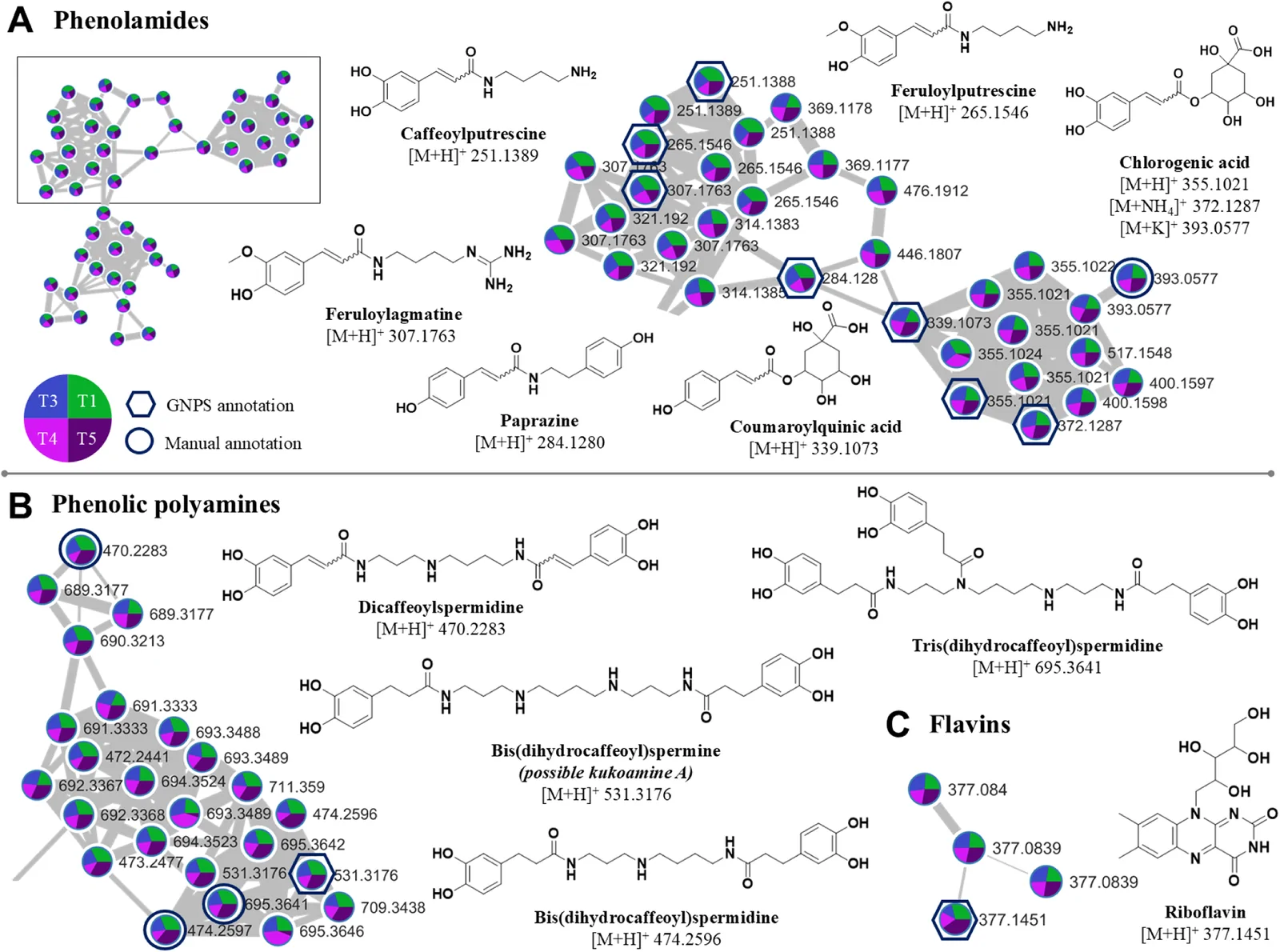
Spectral family of (A) phenolamides and hydroxycinnamate-related metabolites, (B) phenolic polyamines, and (C) flavins obtained by the FBMN tool on the GNPS platform for LC–HRMS data from tomato plants treated with *P. fluorescens* bioinputs.

Other specialized metabolites showed altered abundance levels after the treatments according to FBMN analysis. They include the phenolic polyamines dicaffeoylspermidine [M + H]^+^, bis­(dihydrocaffeoyl)­spermidine [M + H]^+^, bis­(dihydrocaffeoyl)­spermine [M + H]^+^, and tris­(dihydrocaffeoyl)­spermidine [M + H]^+^ (Figure 6B), and the flavin riboflavin [M + H]^+^ (Figure 6C). All annotated metabolites from these spectral families exhibited a decrease in levels with treatments T3 and T4, and riboflavin levels increased in T5.

Several phenolamides, also termed hydroxycinnamic acid amides, and hydroxycinnamates have been reported in *S. lycopersicum*, and their accumulation is commonly associated with plant stress responses and defense against pathogen attack.^63,64^ Plant growth-promoting rhizobacteria (PGPR) can also stimulate the synthesis of compounds of this class, such as chlorogenic acid, improving antioxidant activity.^65^ The maintenance or increase in the levels of many metabolites shown in Figure 5A was observed mainly in T3 and T5, suggesting that foliar application may be associated with upregulation of metabolic pathways involved in phenolamide and hydroxycinnamate biosynthesis.^66^ On the other hand, the reduction in phenolamide levels observed mainly in T4 may suggest a downregulation of plant metabolic pathways associated with these SMs in response to soil application of the cell-free bioinput. To investigate the relationship between the application type and modulation in the biosynthesis of hydroxycinnamic acid derivatives, additional targeted studies would be necessary.

Although described for *S. lycopersicum*,^67^ the biological functions of the phenolic polyamines described above are poorly understood. Nevertheless, the maintenance of their abundance in T5 may suggest the preservation of important biosynthetic pathways in this treatment. This could potentially contribute to a more stable metabolic balance under evaluated conditions. In contrast, reduced levels of phenolic polyamines were observed in T3 and T4, suggesting possible physiological trade-offs among growth, resource allocation, and specialized metabolism. A similar trend was observed for riboflavin, whose levels were also reduced in T3 and T4. Riboflavin plays a vital role in various metabolic processes, especially in photosynthesis^68^ and is therefore closely associated with plant growth and development. Notably, T3 and T4 were the only treatments that significantly reduced leaf biomass compared to the control, as shown in Figure 2E and F.

Although no direct causal relationship can be established, the modulation of phenolic polyamines and riboflavin in these treatments may be associated with observed physiological alterations. Conversely, the increase in the level of riboflavin detected in T5 may indicate a more preserved metabolic status in plants subjected to this treatment. Consistent with this hypothesis, T5 did not show a statistically significant reduction in leaf biomass in the agronomic analyses. Taken together, these findings suggest that foliar application of the cell-free bioinput derived from *P. fluorescens* ATCC_13525 promoted a distinct modulation of plant-specialized metabolism, which could potentially contribute to the maintenance of plant performance under the evaluated application conditions.

## Conclusions

In this study, two bioinputs with active and inactive *P. fluorescens* ATCC_13525 cells were evaluated in terms of metabolic composition and the effects they promoted in the Micro-Tom plant model under controlled cultivation conditions. Untargeted metabolomics analysis of the bioinputs led to the annotation of 12 microbial specialized metabolites whose abundance changed among the products. They included metabolites of different classes and multiple biological functions described, mostly related to plant signaling and growth regulation. Plant treatments exhibited better results for root biomass and fruit quality parameters. Soil treatment with the cell-free product (T4) led to an increase in Mo concentrations in leaves and fruits, whereas leaf spray with the product containing active bacterial cells (T3) increased calcium concentration in fruits. Soil and foliar applications of the cell-free bioinput (T4 and T5, respectively) stimulated root growth and increased °Brix and phosphorus concentration. On the other hand, all treatments negatively affected plant reproductive performance, resulting in reduced fruit yield. Only T5 did not show significant differences in ripe fruit mass compared to the control. Likewise, gas exchange analyses revealed no enhancement in photosynthetic performance or alterations in plant water-use status. In contrast, plant metabolomic analysis exhibited significant changes in specialized metabolism, particularly in T4 and T5, including modulations in indoles, flavonoids, glycerophospholipids, flavins, and some phenolamides. Collectively, these findings indicate that the cell-free bioinput elicited distinct physiological and metabolic responses in Micro-Tom tomato, contributing to improved vegetative growth and selected fruit quality traits, likely through the direct action of bioactive metabolites present in the formulation. However, these beneficial effects were accompanied by a reduction in productivity, indicating an imbalance in resource allocation between vegetative growth and fruit production. Overall, our results highlight the complexity of plant responses to *Pseudomonas fluorescens* based-bioinputs and provide new insights into how formulations differing in bacterial cell viability influence plant physiology and metabolism, supporting the development of safer and more efficient microbial bioinputs in sustainable agriculture.

## Materials and Methods

### Bacterial Strain and Bioinput Composition

The bioinputs obtained from the exclusive fermentation of *Pseudomonas fluorescens* ATCC_13525 in yeast extract and glycerol were supplied by Rovensa Next Brazil (Hortolândia, SP, Brazil). The cultivation was conducted in 1 L baffled Erlenmeyer flasks containing 300 mL of the previously described culture medium under orbital agitation (140 rpm) at 30 °C. The formulation with active bacterial cells (10^8^ CFU/mL), commercially marketed by the company, was obtained after approximately 18 h of cultivation, when the culture reached the targeted OD_600_ for transfer. The formulation with inactive bacterial cells was obtained by an additional centrifugation step at 4193*g* for 1 h at 12 °C of the bacterial culture and, subsequently, by filtration through a 0.22 μm vacuum-driven filtration system K15-1500 (Kasvi, Brazil). For metabolite analysis, the samples were stored at 4 °C after collection.

### Plant Material and Treatment Methods

Tomato seeds for the LA3911 accession [*Solanum lycopersicum* L. cv. Micro-Tom (MT)] were obtained from the University of São Paulo repository (www.esalq.usp.br/tomato) and germinated in a 1:1 (v/v) mixture of Basaplant substrate (Arthur Nogueira, SP, Brazil) and expanded vermiculite, supplemented with NPK 10:10:10 (1 g/L) and limestone (4 g/L). Fifteen days after germination, the plants were transferred individually to 430 mL capacity pots containing the same mixture as for sowing, supplemented with NPK 10:10:10 (8 g/L) and limestone (4 g/L). The cultivar was grown in a greenhouse at the Luiz de Queiroz College of Agriculture – USP (Piracicaba, SP, Brazil) under controlled conditions: automatic irrigation four times per day until reaching field capacity, a 12 h/13 h (winter/summer) photoperiod, and a mean temperature of 28 °C. Eleven days after transplanting, the plants were treated with the bioinputs only once via soil or foliar application as outlined in Table 1. A single application was adopted to evaluate the residual effectiveness of each treatment throughout crop development, allowing a direct comparison among treatments without interfering effects of additional applications. For this, bioinputs were diluted in water at 0.29 mL/L and applied only once using a spray volume equivalent to 70 mL/m^2^ for both application methods. Considering the upper opening area of the pots (10.5 cm diameter), the estimated spray volume applied per pot was approximately 0.61 mL of diluted solution. Based on the doses described in Table 1, this corresponded to approximately 0.43 and 0.17 μL of bioinput per pot for soil and foliar treatments, respectively. The soil treatment was carried out near the plant’s base, while the foliar treatment was performed with the soil covered to ensure that the product would only be in contact with the aerial part.

**Table 1:** Treatment Conditions Used in the Development of the Micro-Tom Tomato

Treatment	Description	Region of application	Dosage
T1	Untreated	-	-
T2	Sample with active *P. fluorescens* cells	Soil	0.05 mL/m^2^
T3	Sample with active *P. fluorescens* cells	Foliar	0.02 mL/m^2^
T4	Sample with inactive *P. fluorescens* cells	Soil	0.05 mL/m^2^
T5	Sample with inactive *P. fluorescens* cells	Foliar	0.02 mL/m^2^

### Agronomic Measures

The effects of the treatments on plant development were evaluated by measuring height at different growth stages using 10 biological replicates per treatment (*n* = 10), determining leaf area (*n* = 5), as well as the fresh and dry mass of roots (*n* = 12), and shoot fresh and dry masses (*n* = 11). For leaf area analysis, a planimeter, model LI-3000A (LI-COR Inc., Lincoln, USA) was used. Gas exchange analyses were carried out on fully developed leaves (*n* = 5) at 3, 13, and 23 days after treatment. Net photosynthetic rate (A), stomatal conductance (gs), transpiration rate (E), and Intracellular CO_2_ concentration (Ci) were measured using an infrared gas analyzer (IRGA) model LI-6800 (LI-COR Inc., Lincoln, USA). Measurements were carried out at ambient CO_2_ concentration, photosynthetic photon flux density (PPFD) at 1000 mmol m^–2^ s^–1^, and a temperature of 25.5 ± 0.5 °C. Productivity and quality parameters were evaluated 85 days after sowing by the number of harvested fruits/per plant (*n* = 12), as well as fruit weight and soluble solids content (°Brix) using 22 biological replicates. °Brix was measured using a digital refractometer, PR-101α (Atago, Japan). On the harvest day, leaves and fruits were collected, separated, cleaned, and dried in a forced-air drying oven at 62 °C for 48 h only for nutrient and intrinsic water-use efficiency (*i*WUE) analyses. Carbon isotope composition (δ^13^C) was used to measure *i*WUE (*n* = 4).^69^ For nutrient analysis, the dried leaf and fruit tissues were ground in an electric mill, and the concentrations of the mineral macronutrients nitrogen (N), phosphorus (P), potassium (K), calcium (Ca), magnesium (Mg), and sulfur (S), as well as the micronutrients boron (B), copper (Cu), iron (Fe), manganese (Mn), zinc (Zn), nickel (Ni), and molybdenum (Mo), were determined following previously established procedures for plant matrices using 4 biological replicates per treatment. N was mineralized by sulfuric acid digestion and quantified using the micro-Kjeldahl method. The remaining nutrients were mineralized by nitroperchloric acid digestion, and the extracts were analyzed by colorimetry (P), flame photometry (K), atomic absorption spectrophotometry (Ca, Mg, Mn, Zn, Fe, Cu, and Ni), turbidimetry (S), the incineration method (B), and spectrophotometry following thiocyanate complex formation for Mo.

### Metabolite Extraction

Metabolite extraction from bioinputs (30 mL) was performed using ice-cold EtOAc at a 2:1 ratio (product:solvent, v/v). After solvent addition, the mixture was sonicated for 30 min and centrifuged at 15557*g* for 5 min at 4 °C. This process was repeated three times, and the supernatants were gathered. The resulting extract was treated with Na_2_SO_4(s)_, filtered, and concentrated under reduced pressure. Four biological replicates were prepared, and the corresponding culture medium for each product was used as the control. For leaf metabolite extraction, the first fully expanded leaf was harvested 10 days after treatment to ensure that the analyzed leaves had developed after bioinput application and had not been directly exposed to the foliar spray, minimizing interference from residual compounds from the applied product. Immediately after harvest, the samples were immersed in liquid nitrogen and stored at −80 °C until use. The fresh leaves were powdered in liquid nitrogen, and the metabolites were extracted as described previously.^12^ Ten individual plants from each treatment were selected randomly for analysis, and the samples were prepared in two analytical replicates.

### Sample Preparation and Instrumental Analysis

The concentrated extracts were weighed, reconstituted in HPLC-grade methanol to a final concentration of 5 mg/mL, and filtered through a 0.22 μm PTFE syringe filter directly into a 2 mL vial for LC–HRMS analysis. Quality control (QC) samples were prepared by pooling equal volumes (15 μL) of all samples involved in the experiment. The samples were maintained at −80 °C until analysis. The LC–HRMS analysis was performed on an Ultra-High Performance Liquid Chromatograph Ultimate 3000 coupled to a Q-Exactive Plus Hybrid Quadrupole-Orbitrap mass spectrometer (Thermo Fisher Scientific Inc., USA) equipped with a heated electrospray ionization source (HESI). Chromatographic separation was performed using a reversed-phase Intensity Solo C18 column (Bruker, USA, 1.8 μm, 2.1 × 100 mm) with solvent A (water) and solvent B (acetonitrile) containing 0.1% (v/v) formic acid. The mobile phase was eluted in a gradient from 5% to 98% B at a flow rate maintained at 250 μL/min. For bioinput analysis, the gradient elution was: 0–2 min: 5% B, 2–10 min: 70% B, 10–13 min: 80% B, 13–15 min: 98% B, 15–16 min: 98% B, 16–17 min: 5% B, 17–20 min: 5% B. For Micro-Tom analysis, the gradient elution was: 0–2 min: 5% B, 2–6 min: 70% B, 6–10 min: 98% B, 10–12 min: 98% B, 12–13 min: 5% B, 13–19 min: 5% B. The injection volume used was 5 μL, and the column temperature was set at 40 °C. Samples were analyzed in fullMS/ddMS^2^ acquisition mode, operating in positive ionization mode, over a scan range from *m*/*z* 100 to 1500 Da with the following parameters: nebulizing gas temperature of 250 °C, nebulization gas pressure of 45 psi, capillary voltage of +3500 V, and normalized collision energy of 33 eV. To monitor the variability of the analytical methods, the QC was analyzed three times at the beginning and end of the batch, and once after every four samples. Within a batch, the samples were randomized.

### Data Processing and Statistical Analysis

Agronomic measures were subjected to one-way analysis of variance (ANOVA) using GraphPad Prism 8.0.1 software (GraphPad Software, USA) to evaluate statistical differences between treatments and their respective controls. Data were expressed as the mean ± standard deviation (SD) in bar graphs. The raw files acquired in the LC–HRMS analyses were converted to .mzXML file format and processed by the mzmine 4.4.3 software (mzio GmbH, Germany). The generated correlation matrix of features versus corresponding peak areas was used to perform multivariate and univariate statistical analyses on the MetaboAnalyst 6.0 platform (https://www.metaboanalyst.ca), with normalization by the median and data scaling by the Pareto method. Outlier removal was based on Principal Component Analysis (PCA) and Random Forest Regression. The discriminating features were selected based on *p*-value ≤0.05, determined using ANOVA with Tukey’s posthoc test or a two-tailed Student’s *t*-test, and variable importance in the projection (VIP) score ≥0.6, adopted as an exploratory criterion to retain variables with moderate discriminatory contribution to treatment discrimination that could still be biologically relevant. Metabolite annotation (identification level 2) was achieved according to the Metabolomics Standards Initiative (MSI) by determining the exact mass of the precursor ion (*m*/*z*), thereby establishing the molecular formula with a mass error ≤5 ppm, and by comparing the MS^2^ fragmentation spectrum with standards in available databases.^70^ The databases include GNPS (https://library.gnps2.org/), MassBank.eu (https://massbank.eu/MassBank/), and MoNA (https://mona.fiehnlab.ucdavis.edu/). Also, the relative abundance of specific ions was measured by the chromatographic peak area from the extracted ion chromatogram (EIC) in all replicates.

### Data Analysis

Metabolic dereplication and molecular networking (MN) were performed using the Global Natural Products Social Molecular Networking platform (GNPS), based on the similarity of fragmentation spectra. The .mgf and .csv files obtained from data processing in the mzmine 4.4.3 software, and a metadata table in .txt file format, were submitted to GNPS using the Feature-Based Molecular Networking (FBMN) method.^71^ The FBMN job link is available at https://gnps2.org/status?task=0d90197d23914d80820b3a5ac957e0d8. Molecular networking was obtained considering precursor-ion and fragment-ion mass tolerances of ±0.02 Da to create consensus spectra. The connections between nodes (lines) were formed with a minimum cosine value equal to 0.7, referring to the similarity between two MS/MS spectra on a scale from 0 to 1, and a minimum of 3 fragment ions in common. Simultaneously, experimental spectra were compared with GNPS databases using the same parameters as the input data. Thus, the correspondence between the experimental and standard MS/MS spectra to be considered a molecular annotation (hit) necessarily needed to have a consensus value greater than or equal to 0.7 and at least 3 identical fragments. The visualization and editing of the molecular networks were performed using the Cytoscape 3.10.2 software (Cytoscape Consortium, USA). In silico data analyses were performed using SIRIUS 5.8.6 software^72^ from the .mgf files. The accuracy of structural predictions was assessed by the Confidence Of Small Molecule IdentifiCations (COSMIC) method.^73^ The results were visualized in the software itself.

## Supplementary Material



## Notes

Figure S1: MicrobeMASST results showing the detection of 8-oxo-isopentenyladenine and cyclo­(Trp–Pro) in *P. fluorescens* MS/MS data sets deposited in the GNPS database; Figure S2: macro and micronutrient content in leaves treated with the bioinputs from *P. fluorescens*; Figure S3: intrinsic water-use efficiency (*i*WUE) of tomato plants treated with the bioinputs from *P. fluorescens;* Figure S4: representative seedlings and roots from treatments T1 to T5; Figure S5: macro and micronutrient content in fruits treated with the bioinputs from *P. fluorescens*; Table S1: differential specialized metabolites between the two bioinputs from *P. fluorescens* ATCC_13525; Table S2: discriminant metabolites in micro-Tom leaf samples treated with bioinputs from *P. fluorescens* ATCC_13525 (PDF)
