# Supplementary material for: Integrative Metabolomic and Agronomic Analyses Reveal the Impact of Pseudomonas fluorescens-Based Bioinputs on the Micro-Tom Tomato (Solanum lycopersicum) Model System
Source: ACS Omega. 2026 Sep 14;11(38):57072–84. doi: 10.1021/acsomega.6c04589 (PMC13629025; doi:10.1021/acsomega.6c04589)
Supplement: Supplementary file 1 [file ao6c04589_si_001.pdf]

Supporting Information

Integrative metabolomic and agronomic analyses reveal the impact of *Pseudomonas fluorescens*-based bioinputs on the Micro-Tom tomato (*Solanum lycopersicum*) model system

Stephanie Nemesio da Silva [a], Rodrigo Alberto Repke [b], Lázaro Eustaquio Pereira Peres [c], Alice Aranda Peres [c], Rafael Costa Santos Rocha [b], Danilo da Cruz Centeno [d], Johana Rincones Pérez [b] and Taicia Pacheco Fill [a,e*]

*Corresponding author.

E-mail: taicia@unicamp.br (T. P. Fill)

Contents

Page#

S2 Figure S1. MicrobeMASST results showing the detection of A) 8-oxo-isopentenyladenine and its corresponding B) Experimental MS/MS spectrum and C) Matching repository MS/MS spectrum, as well as D) Cyclo(Trp–Pro) and its corresponding E) Experimental MS/MS spectrum and F) Matching repository MS/MS spectrum in *P. fluorescens* MS/MS datasets deposited in the GNPS database

S4 Figure S2. Macro and micronutrient content in leaves treated with the bioinputs from *P. fluorescens*

S5 Figure S3. Intrinsic water-use efficiency (*i*WUE) of tomato plants treated with the bioinputs from *P. fluorescens*.

S5 Figure S4. Representative seedlings and roots from treatments T1 to T5.

S6 Figure S5. Macro and micronutrient content in fruits treated with the bioinputs from *P. fluorescens*

S7 Table S1. Differential specialized metabolites between the two bioinputs from *P. fluorescens* ATCC_13525

S9 Table S2. Discriminant metabolites in Micro-Tom leaf samples treated with bioinputs from *P. fluorescens* ATCC_13525

A

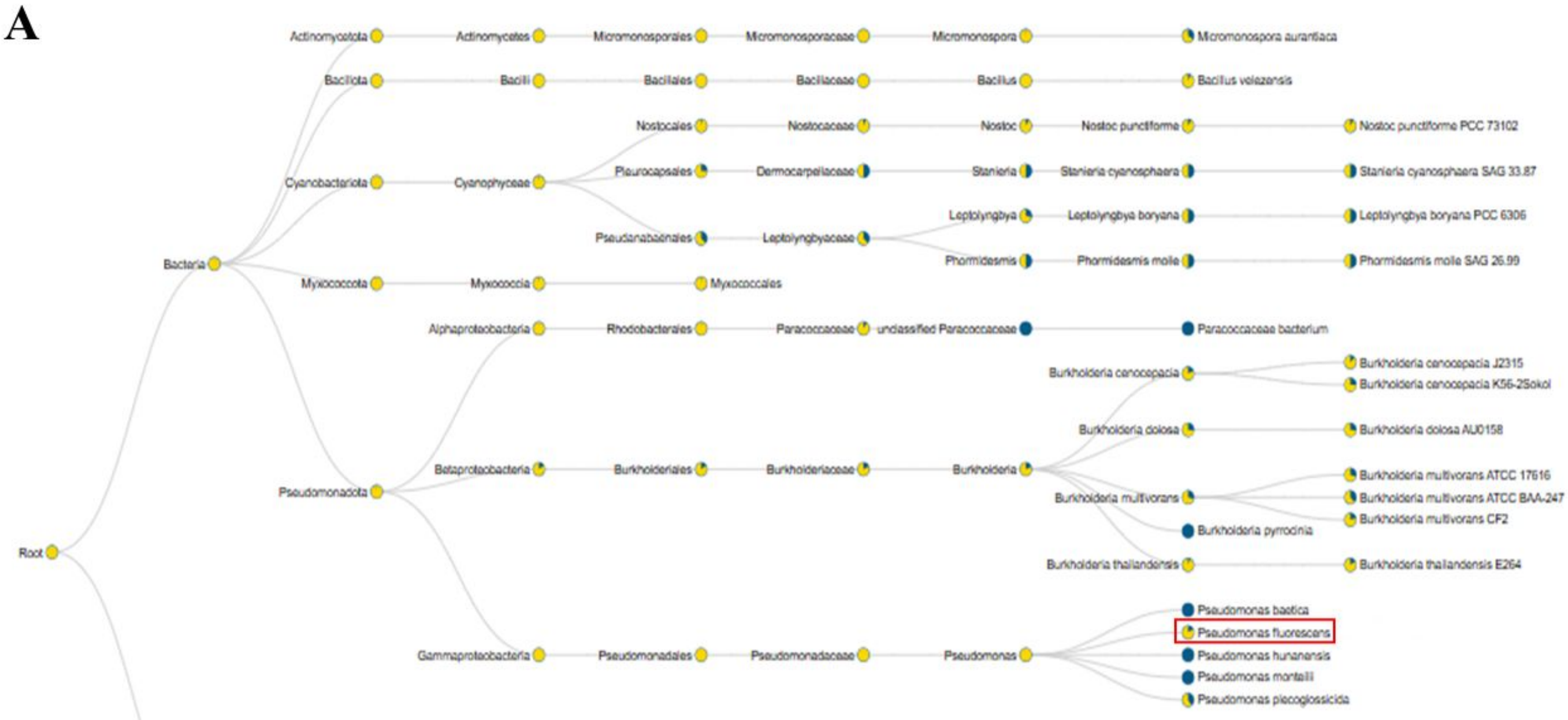
Root
Bacteria
Actinomycetota
Actinomycetes
Micromonosporales
Micromonosporaceae
Micromonospora
Micromonospora aurantiaca
Bacillota
Bacilli
Bacillales
Bacillaceae
Bacillus
Bacillus velezensis
Cyanobacteriota
Cyanophyceae
Nostocales
Nostocaceae
Nostoc
Nostoc punctiforme
Nostoc punctiforme PCC 73102
Pleurocapsales
Dermocarpellaceae
Stanieria
Stanieria cyanosphaera
Stanieria cyanosphaera SAG 33.87
Pseudanabaenales
Leptolyngbyaceae
Leptolyngbya
Leptolyngbya boryana
Leptolyngbya boryana PCC 6306
Phormidesmis
Phormidesmis molle
Phormidesmis molle SAG 26.99
Myxococcota
Myxococcia
Myxococcales
Pseudomonadota
Alphaproteobacteria
Rhodobacterales
Paracoccaceae
unclassified Paracoccaceae
Paracoccaceae bacterium
Betaproteobacteria
Burkholderiales
Burkholderiaceae
Burkholderia
Burkholderia cenocepacia
Burkholderia cenocepacia J2315
Burkholderia cenocepacia K56-2Sokol
Burkholderia dolosa
Burkholderia dolosa AU0158
Burkholderia multivorans
Burkholderia multivorans ATCC 17616
Burkholderia multivorans ATCC BAA-247
Burkholderia multivorans CF2
Burkholderia pyrrocinia
Burkholderia thailandensis
Burkholderia thailandensis E264
Gammaproteobacteria
Pseudomonadales
Pseudomonadaceae
Pseudomonas
Pseudomonas baetica
Pseudomonas fluorescens
Pseudomonas hunanensis
Pseudomonas montellii
Pseudomonas plecoglossicida

B

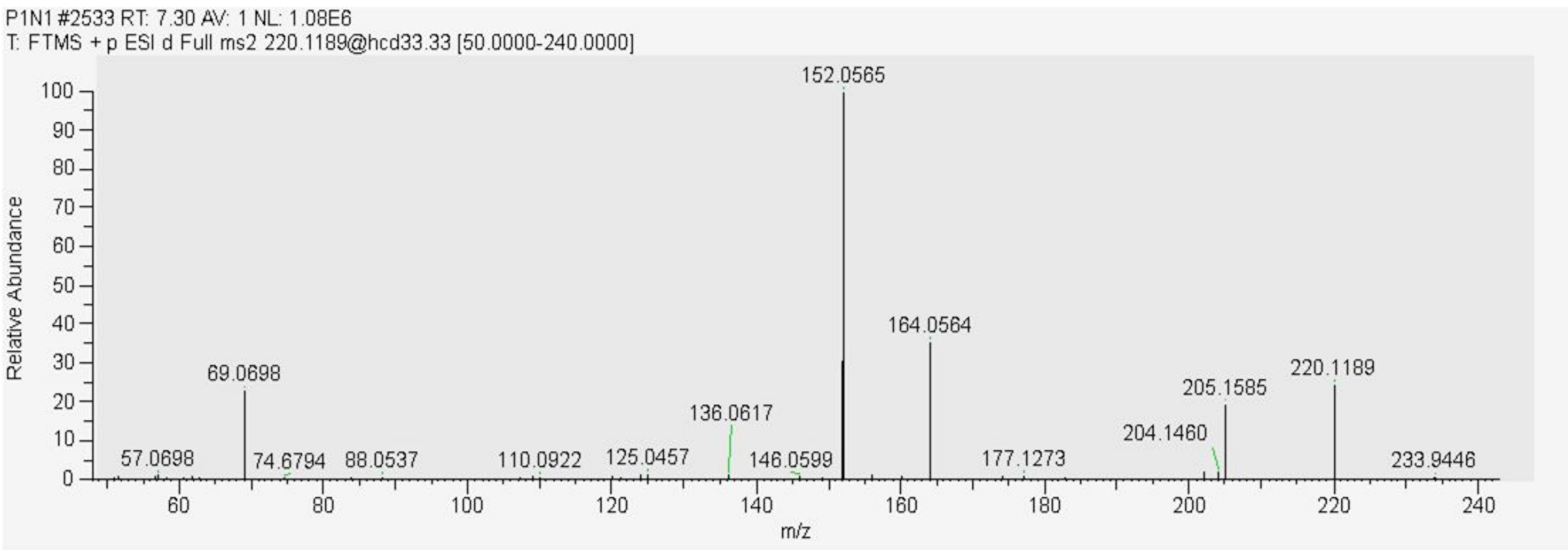
P1N1 #2533 RT: 7.30 AV: 1 NL: 1.08E6
T: FTMS + p ESI d Full ms2 220.1189@hcd33.33 [50.0000-240.0000]
Relative Abundance
m/z
152.0565
164.0564
220.1189
205.1585
204.1460
69.0698
136.0617
57.0698
74.6794
88.0537
110.0922
125.0457
146.0599
177.1273
233.9446

C

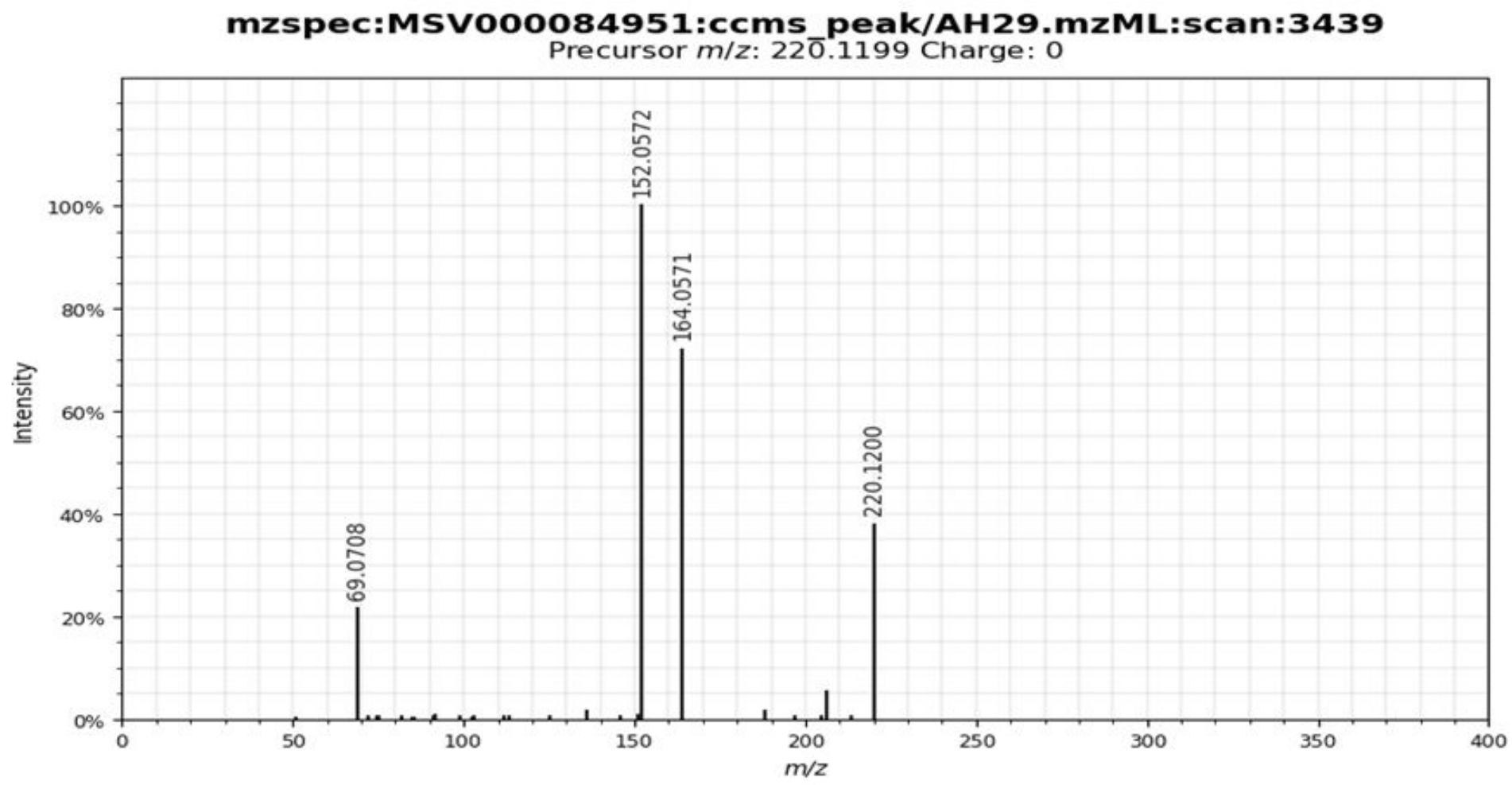
mzspec:MSV000084951:ccms_peak/AH29.mzML:scan:3439
Precursor m/z: 220.1199 Charge: 0
Intensity
m/z
152.0572
164.0571
220.1200
69.0708

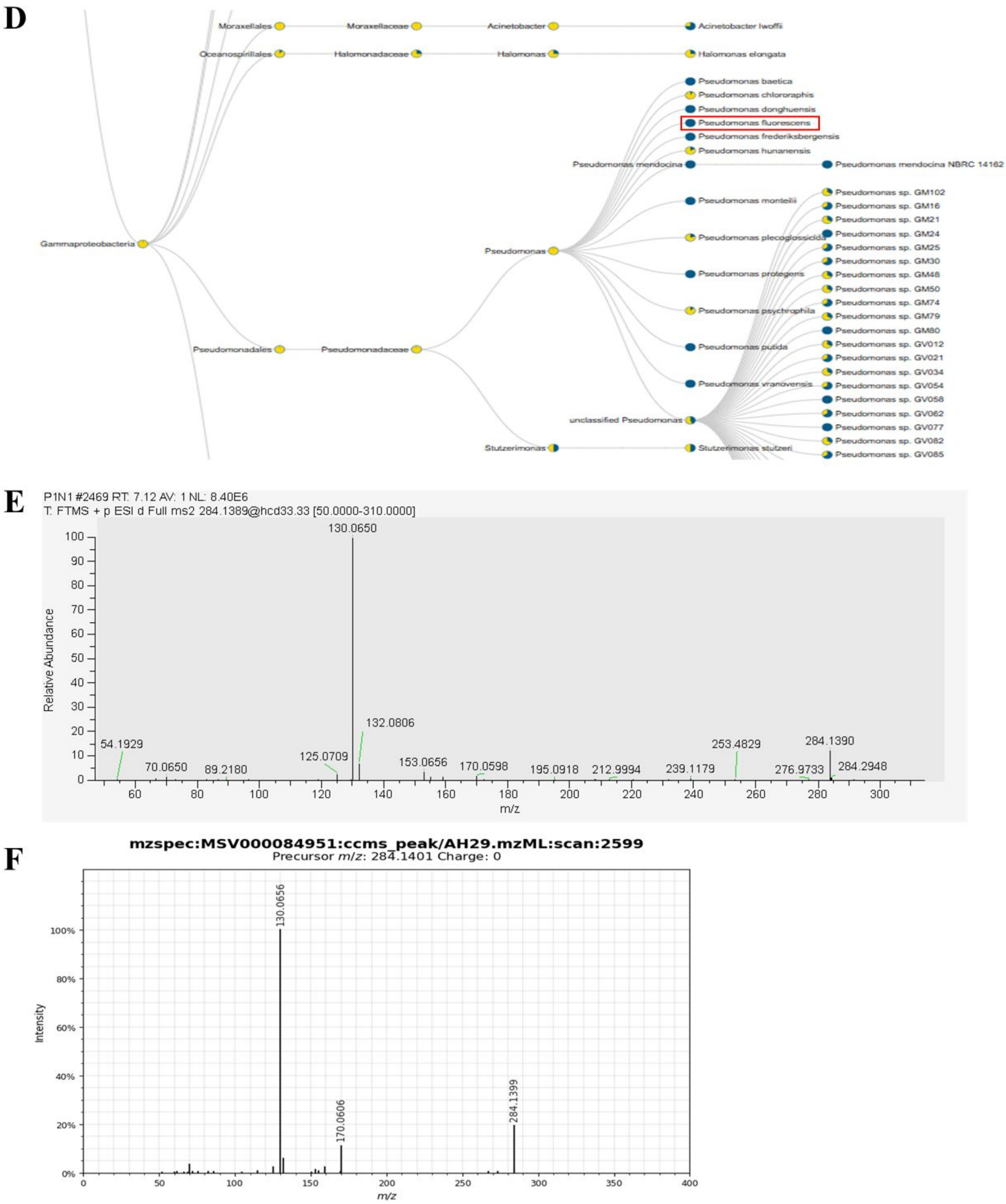

Fig. S1 MicrobeMASST results showing the detection of A) 8-oxo-isopentenyladenine and its corresponding B) Experimental MS/MS spectrum and C) Matching repository MS/MS spectrum, as well as D) Cyclo(Trp–Pro) and its corresponding E) Experimental MS/MS spectrum and F) Matching repository MS/MS spectrum in *P. fluorescens* MS/MS datasets deposited in the GNPS database.

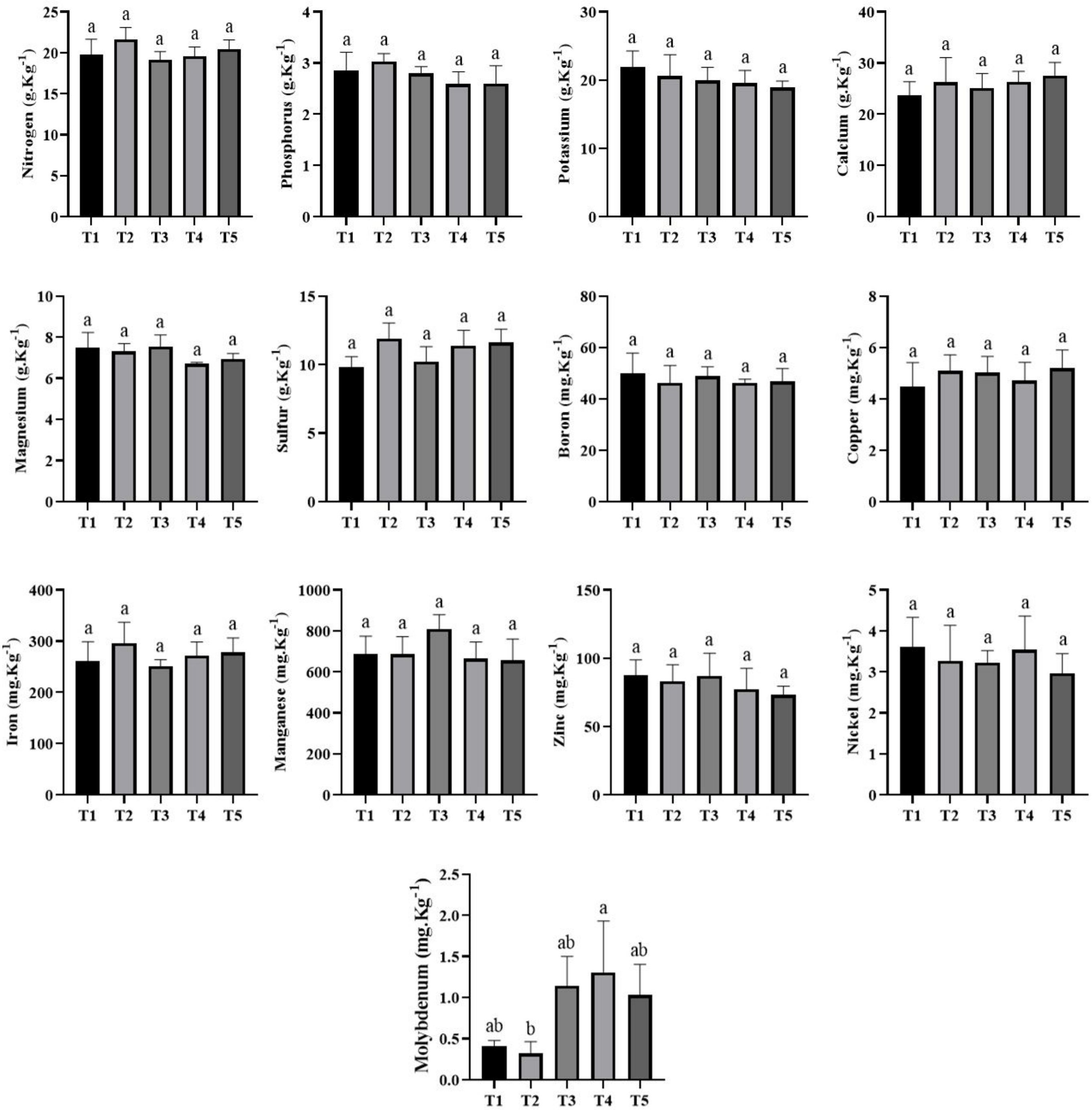

Fig. S2 Macro and micronutrient content in leaves treated with the bioinputs from *P. fluorescens*. Error bars represent the SD (n = 4). Different letters indicate significantly different values at $p \leq 0.05$ (one-way ANOVA, Tukey's post-hoc test).

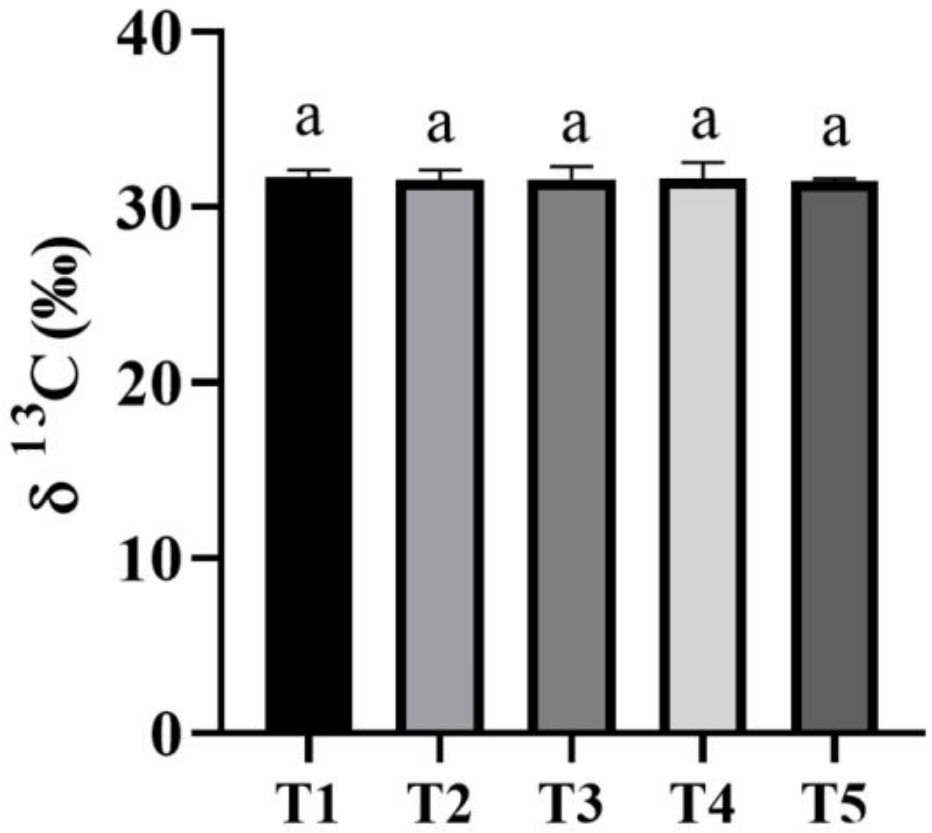

Fig. S3 Intrinsic water-use efficiency (*i*WUE) of tomato plants treated with the bioinputs from *P. fluorescens*. Error bars represent the SD. Different letters indicate significantly different values at $p \leq 0.05$ (one-way ANOVA, Tukey's post-hoc test).

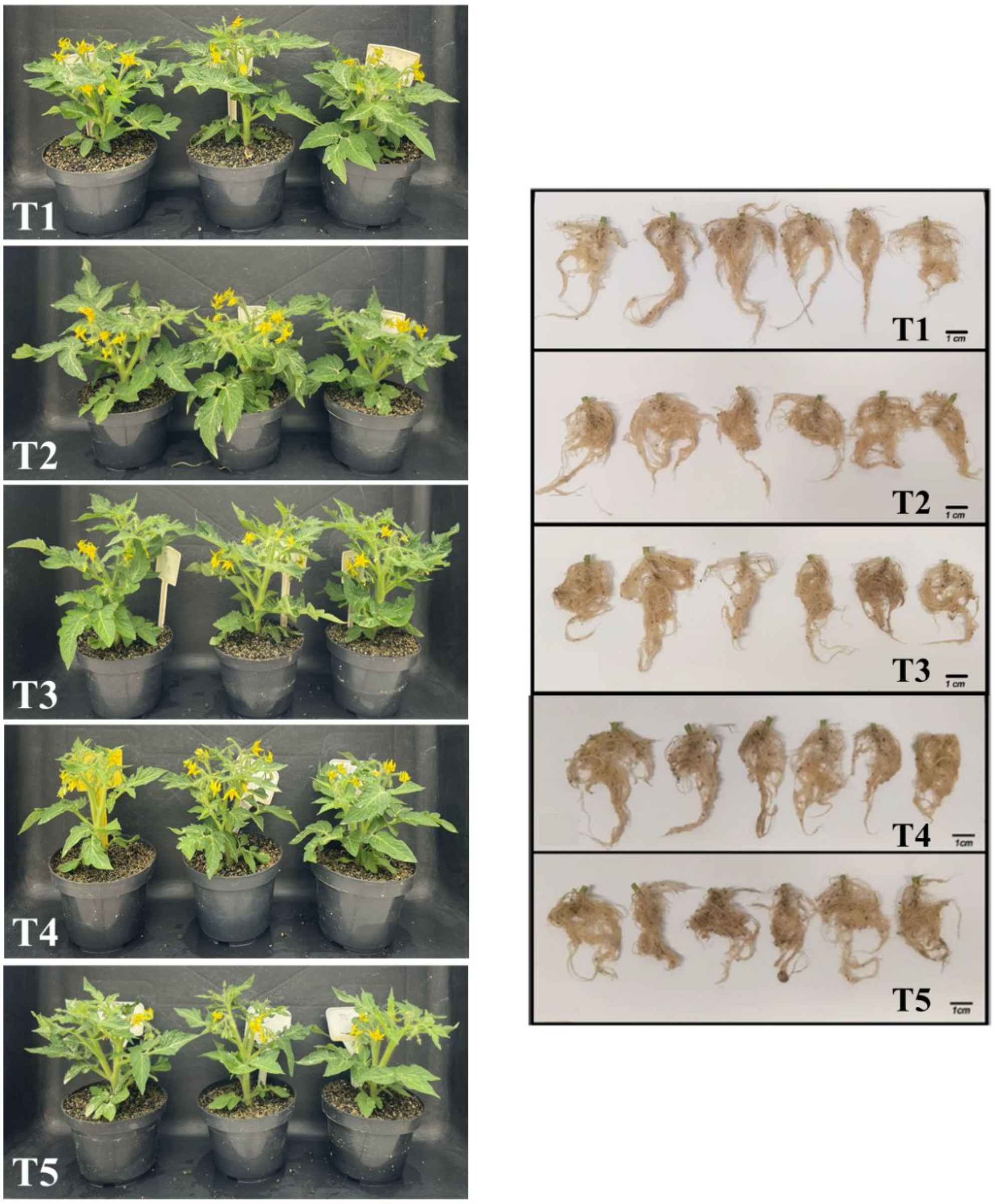

Fig. S4 Representative seedlings and roots from treatments T1 to T5.

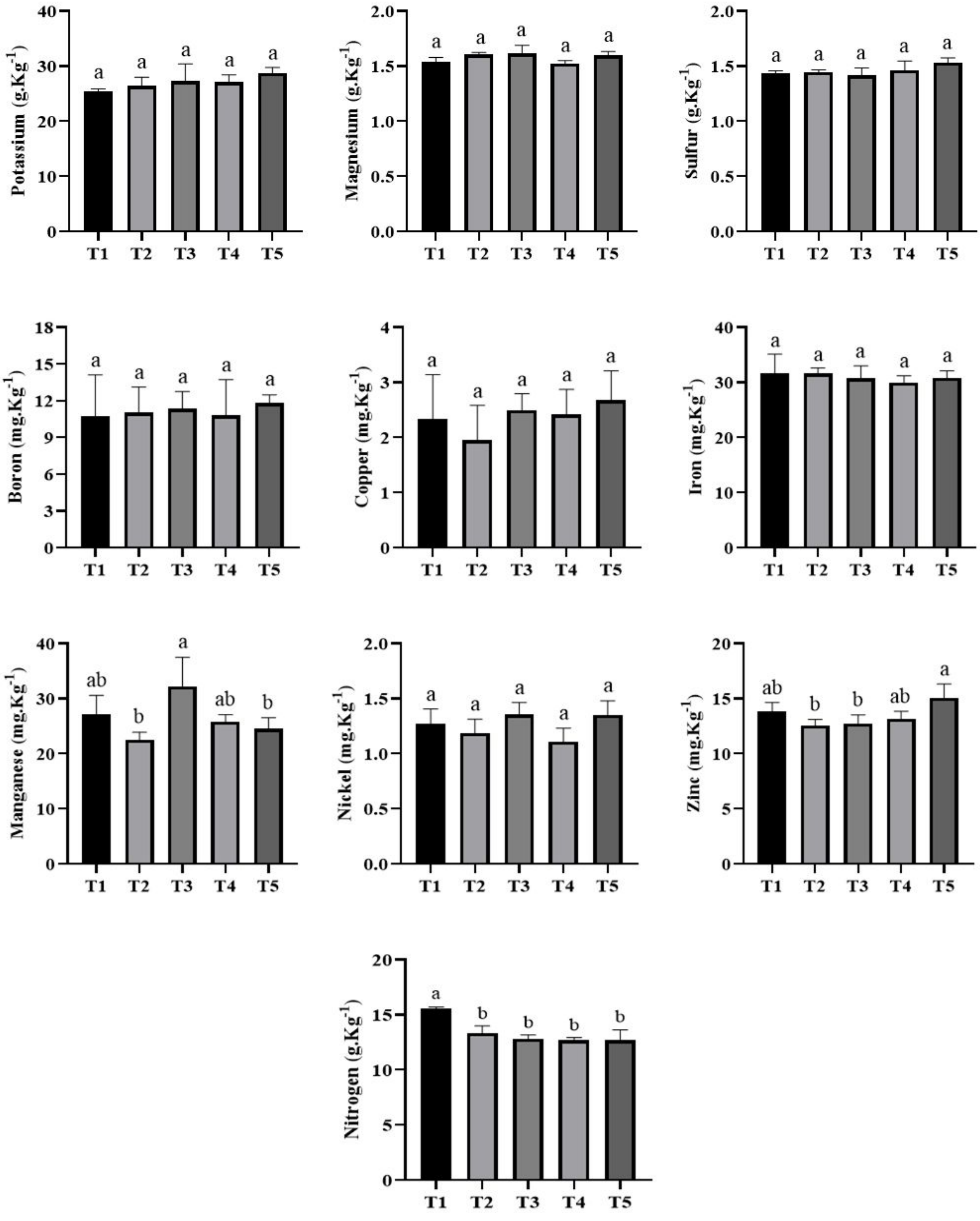

Fig. S5 Macro and micronutrient content in fruits treated with the bioinputs from *P. fluorescens*. Error bars represent the SD (n = 4). Different letters indicate significantly different values at $p \leq 0.05$ (one-way ANOVA, Tukey's post-hoc test).

Table S1. Differential specialized metabolites between the two bioinputs from *P. fluorescens* ATCC_13525.

Chemical Class	Predicting compound	Pubchem CID	p-value	VIP scores	Log2(FC)	Ion formula	Exp. *m/z*	Error (ppm)	RT (min)	MS^2 main fragments	Reference/ Accession ID
Alkylthiols	Acetyl-pantetheine[c]	101639605	0.00209	0.85	-	$[C_{13}H_{24}N_2O_5S+H]^+$	321.1474	-2.13	6.48	114.0548, 149.0741, **191.0845**, 303.1368	[3]
Aminopurines	Isopentenyladenine[a]	92180	$3.27E^{-5}$	3.48	-1.42	$[C_{10}H_{13}N_5+H]^+$	204.1244	-1.11	6.51	69.0698, **136.0616**, 148.0615, 204.1241	CCMSLIB 00000578065 (GNPS)
Aminopurines	Zeatin[a]	449093	$1.44E^{-7}$	1.03	-4.34	$[C_{10}H_{13}N_5O+H]^+$	220.1189	-1.84	5.10	**136.0616**, 148.0616, 185.0820, 202.1084	CCMSLIB 00016151473 (GNPS)
Aminopurines	8-oxo-isopentenyladenine[c]	101408508	$1.02E^{-5}$	0.79	-1.90	$[C_{10}H_{13}N_5O+H]^+$	220.1189	-1.70	7.30	69.0698, 136.0615, **152.0565**, 164.0564	[2]
Diketopiperazines	Cyclo(Trp-Pro)[a]	181567	0.0207	2.82	-2.91	$[C_{16}H_{17}N_3O_2+H]^+$	284.1389	-1.54	7.13	125.0709, **130.0650**, 153.0556, 284.1390	CCMSLIB 00000007122 (GNPS)
Indoles	Isatin[a]	7054	0.00158	3.82	3.75	$[C_8H_5NO_2+H]^+$	148.0390	-1.84	7.34	92.0494, 120.0443, 130.0286, **148.0391**	MSBNK-Fiocruz-FIO00511 (MassBank.eu)
Indoles	Indole-3-acetaldehyde[b]	800	$1.60E^{-5}$	1.01	-	$[C_{10}H_9NO+H]^+$	160.0754	-1.66	8.15	91.0541, 118.0649, 132.0806, **160.0754**	CCMSLIB 00005884893 (GNPS)

Indoles	Indole-3-carboxylic acid[a]	69867	-	0.64	-	$[C_9H_7NO_2+H]^+$	162.0547	-1.82	7.66	**118.0649**, 134.0599, 144.0441, 162.0546	CCMSLIB 00003136454 (GNPS)
Indoles	Indole-3-acetic acid[a]	802	0.000198	-	1.97	$[C_{10}H_9NO_2+H]^+$	176.0704	-1.32	7.29	103.0540, 118.0649, **130.0649**, 158.0598	CCMSLIB 00012176433 (GNPS)
Indoles	N-acetyl-oxotryptamine[c]	4188168	0.000106	1.95	-	$[C_{12}H_{12}N_2O_2+H]^+$	217.0968	-1.41	7.25	**100.0391**, 118.0649, 175.0863, 199.0864	[1]
Purines	6-dimethylaminopurine[a]	3134	$3.76E^{-7}$	3.65	-1.76	$[C_7H_9N_5+H]^+$	164.0928	-1.82	2.34	94.0400, 121.0507, 149.0695, **164.0928**	CCMSLIB 00012348533 (GNPS)
Quinolines	Quinoline-4-carboxylic acid[a]	10243	$2.49E^{-5}$	0.82	6.21	$[C_{10}H_7NO_2+H]^+$	174.0548	-1.01	5.75	128.0492, 146.0595, 156.0441, **174.0548**	CCMSLIB 00010118766 (GNPS)

Annotation basis: (**a**) Experimental MS/MS spectrum matched to reference spectra through data dereplication using the GNPS platform, (**b**) SIRIUS-assisted candidate prediction followed by manual comparison of the experimental MS/MS fragmentation pattern with public spectral databases, and (**c**) SIRIUS-assisted candidate prediction followed by manual comparison of the experimental MS/MS fragmentation pattern with fragmentation data previously reported in the literature.

Table S2. Discriminant metabolites in Micro-Tom leaf samples treated with bioinputs from *P. fluorescens* ATCC_13525.

Chemical class	Predicted compound	Pubchem CID	p-value	VIP scores	Ion formula	Exp. *m/z*	Error (ppm)	RT (min)	MS^2 main fragments	Reference/Accession ID
Amino acids	Glutamine[b]	5961	$5.39E^{-5}$	5.94	$[C_5H_{10}N_2O_3+H]^+$	147.0764	0.09	0.95	**84.0444**, 85.0284, 102.0549, 130.0499	MSBNK-BGC_Munich-RP001301 (MassBank.eu)
Amino acids	Tryptophan[b]	6305	$2.19E^{-13}$<br>$1.83E^{-13}$	5.24<br>5.95	$[C_{11}H_{12}N_2O_2+H]^+$<br>$[C_{11}H_{12}N_2O_2+H-H_3N]^+$	205.0971<br>188.0706	-0.16<br>-0.18	3.56	118.0651, 144.0806, 146.0598, **188.0705**	MSBNK-Antwerp _Univ-METOX _P100401_F638 (MassBank.eu)
Cinnamate ester	Coumaroylquinic acid[b,*]	9945785	-	0.677	$[C_{16}H_{18}O_8+H]^+$	339.1075	0.20	4.85	91.0542 119.0491, **147.0440**, 165.0549	VF-NPL-QEHF006318 (MoNA)
Cinnamate ester	Chlorogenic acid[a,*]	1794427	-	5.34	$[C_{16}H_{18}O_9+H]^+$<br>$[M+NH_4]^+$<br>$[M+K]^+$	355.1021<br>372.1287<br>393.0577	-0.61	4.53	117.0335, 135.0440, 145.0283, **163.0388**	CCMSLIB00004684185 (GNPS)
Flavins	Riboflavin[a,*]	493570	-	-	$[C_{17}H_{20}N_4O_6+H]^+$	377.1451	-1.11	4.51	172.0867, **243.0874**, 359.1343, 377.1451	CCMSLIB00010112847 (GNPS)
Flavonoids	Kaempferol 3-neoherperidoside[a]	5318761	-	3.58	$[C_{27}H_{30}O_{15}+H]^+$	595.1648	-1.60	5.08	71.0491, 85.0284, **287.0547**, 449.1078	CCMSLIB00012335995 (GNPS)
Glycerophospoethanolamines	LPE 18:2[a]	52925130	$1.04E^{-8}$	1.90	$[C_{23}H_{44}NO_7P+H]^+$	478.2926	-0.38	8.54	216.0630, 306.2791, **337.2734**, 460.2820	CCMSLIB00010065601 (GNPS)

Glycerophospholipids	Glycerylphosphorylcholine[a]	11234	0.00378<br>0.0128	3.67<br>2.97	$[C_8H_{20}NO_6P+H]^+$<br>$[C_8H_{20}NO_6P+H-C_3H_6O_2]^+$	258.1100<br>184.0734	-0.09<br>0.32	0.97	**104.1070**, 124.9999, 184.0734, 258.1102	CCMSLIB00006679241 (GNPS)
Indoles	Lycoperodine-1/ Cyclomethyltryptophan[c]	449440	$4.82E^{-8}$	0.76	$[C_{12}H_{12}N_2O_2+H]^+$	217.0971	-0.29	4.25	130.0500, **144.0808**, 200.1283 217.0965	[4]
Lysophosphatidylcholine	LPC 18:2[a]	11005824	$1.88E^{-6}$	1.98	$[C_{26}H_{50}NO_7P+H]^+$	520.3397	-0.08	8.58	104.1070, 124.9998, **184.0733**, 502.3279	CCMSLIB00010099321 (GNPS)
Phenolamides	N-caffeoylputrescine[a,*]	5280559	0.00287	5.66	$[C_{13}H_{18}N_2O_3+H]^+$	251.1389	-0.59	1.38	89.1073, **163.0388**, 234.1122, 251.1387	CCMSLIB00005739860 (GNPS)
Phenolamides	Feruloylputrescine[a,*]	5281796	0.00869	1.27	$[C_{14}H_{20}N_2O_3+H]^+$	265.1546	-0.37	2.34	72.0808, 145.0283, **177.0546**, 265.1549	CCMSLIB00005748443 (GNPS)
Phenolamides	Paprazine[a,*]	5372945	-	0.85	$[C_{17}H_{17}NO_3+H]^+$	284.1280	0.08	5.71	93.0699, 121.0648, **147.0439**, 284.1279	CCMSLIB00006405612 (GNPS)
Phenolamides	Feruloylagmatine[b,*]	46173376	-	2.58	$[C_{15}H_{22}N_4O_3+H]^+$	307.1763	-0.64	3.59	145.0283, **177.0546**, 290.1496, 307.1762	PR311014 (MoNA)
Phenolic polyamines	Dicaffeoylspermidine[b,*]	102138255	-	-	$[C_{25}H_{31}N_3O_6+H]^+$	470.2285	-0.034	3.97	163.0388, 220.0966,	MSBNK-mFam-MC03_000086

									308.1966, **470.2283**	(MassBank.eu)
Phenolic polyamines	N[1],N[10]-bis(dihydrocaffeoyl) spermidine[c,*]	15730308	0.0222	0.14	$[C_{25}H_{35}N_3O_6+H]^+$	474.2595	-0.72	3.95	165.0544, **222.1122**, 310.2119, 474.2594	[5,6]
Phenolic polyamines	Bis(dihydrocaffeoyl)spermine[a,*] (possible kukoamine A)	5318865	0.000764	0.13	$[C_{28}H_{42}N_4O_6+H]^+$	531.3176	-0.16	1.03	165.0544, **222.1123**, 293.1857, 531.3176	CCMSLIB00006435219 (GNPS)
Phenolic polyamines	N[1],N[5],N[14]-tris (dihydrocaffeoyl)spermine[c,*]	101723031	0.0112	1.18	$[C_{37}H_{50}N_4O_9+H]^+$	695.3641	-1.32	4.35	165.0546, **222.1123**, 531.3165, 695.3644	[5,6]

Annotation basis: (**a**) Experimental MS/MS spectrum matched to reference spectra through data dereplication using the GNPS platform, (**b**) SIRIUS-assisted candidate prediction followed by manual comparison of the experimental MS/MS fragmentation pattern with public spectral databases, and (**c**) SIRIUS-assisted candidate prediction followed by manual comparison of the experimental MS/MS fragmentation pattern with fragmentation data previously reported in the literature. (*****) Metabolites exhibited in the molecular networking analysis.

References

(1) Cheng, C.; MacIntyre, L.; Abdelmohsen, U. R.; Horn, H.; Polymenakou, P. N.; Edrada-Ebel, R.; Hentschel, U. Biodiversity, Anti-Trypanosomal Activity Screening, and Metabolomic Profiling of Actinomycetes Isolated from Mediterranean Sponges. *PLoS ONE* **2015**, *10* (9), e0138528. https://doi.org/10.1371/journal.pone.0138528.

(2) Tarkowski, P.; Floková, K.; Václavíková, K.; Jaworek, P.; Raus, M.; Nordström, A.; Novák, O.; Doležal, K.; Šebela, M.; Frébortová, J. An Improved in Vivo Deuterium Labeling Method for Measuring the Biosynthetic Rate of Cytokinins. *Molecules* **2010**, *15* (12), 9214–9229. https://doi.org/10.3390/molecules15129214.

(3) Thapa, B. B.; Huo, C.; Budhathoki, R.; Chaudhary, P.; Joshi, S.; Poudel, P. B.; Magar, R. T.; Parajuli, N.; Kim, K. H.; Sohng, J. K. Metabolic Comparison and Molecular Networking of Antimicrobials in Streptomyces Species. *IJMS* **2024**, *25* (8), 4193. https://doi.org/10.3390/ijms25084193.

(4) Sai Sachin, L.; Nagarjuna Chary, R.; Pavankumar, P.; Prabhakar, S. Identification and Characterization of Reaction Products of 5-Hydroxytryptamine with Methylglyoxal and Glyoxal by Liquid Chromatography/Tandem Mass Spectrometry. *Rapid Communications in Mass Spectrometry* **2018**, *32* (17), 1529–1539. https://doi.org/10.1002/rcm.8195.

(5) Li, Y.-Y.; Wang, H.; Zhao, C.; Huang, Y.-Q.; Tang, X.; Cheung, H.-Y. Identification and Characterization of Kukoamine Metabolites by Multiple Ion Monitoring Triggered Enhanced Product Ion Scan Method with a Triple-Quadruple Linear Ion Trap Mass Spectrometer. *J. Agric. Food Chem.* **2015**, *63* (50), 10785–10790. https://doi.org/10.1021/acs.jafc.5b04321.

(6) Xiao, X.; Ren, W.; Zhang, N.; Bing, T.; Liu, X.; Zhao, Z.; Shangguan, D. Comparative Study of the Chemical Constituents and Bioactivities of the Extracts from Fruits, Leaves and Root Barks of Lycium Barbarum. *Molecules* **2019**, *24* (8), 1585. https://doi.org/10.3390/molecules24081585.
